# Ovaries of estrogen receptor 1-deficient mice show iron overload and signs of aging

**DOI:** 10.3389/fendo.2024.1325386

**Published:** 2024-02-23

**Authors:** Sarah K. Schröder, Marinela Krizanac, Philipp Kim, Jan C. Kessel, Ralf Weiskirchen

**Affiliations:** Institute of Molecular Pathobiochemistry, Experimental Gene Therapy and Clinical Chemistry (IFMPEGKC), Rheinisch-Westfälische Technische Hochschule (RWTH) University Hospital Aachen, Aachen, Germany

**Keywords:** estrogen receptor alpha, ERα, *Esr1*, ovary, iron, macrophage, multinucleated giant cells, aging

## Abstract

**Introduction:**

Estrogens are crucial regulators of ovarian function, mediating their signaling through binding to estrogen receptors. The disruption of the estrogen receptor 1 (*Esr1*) provokes infertility associated with a hemorrhagic, cystic phenotype similar to that seen in diseased or aged ovaries. Our previous study indicated the possibility of altered iron metabolism in *Esr1*-deficient ovaries showing massive expression of lipocalin 2, a regulator of iron homeostasis.

**Methods:**

Therefore, we examined the consequences of depleting *Esr1* in mouse ovaries, focusing on iron metabolism. For that reason, we compared ovaries of adult *Esr1*-deficient animals and age-matched wild type littermates.

**Results and discussion:**

We found increased iron accumulation in *Esr1*-deficient animals by using laser ablation inductively coupled plasma mass spectrometry. Western blot analysis and RT-qPCR confirmed that iron overload alters iron transport, storage and regulation. In addition, trivalent iron deposits in form of hemosiderin were detected in *Esr1*-deficient ovarian stroma. The depletion of *Esr1* was further associated with an aberrant immune cell landscape characterized by the appearance of macrophage-derived multinucleated giant cells (MNGCs) and increased quantities of macrophages, particularly M2-like macrophages. Similar to reproductively aged animals, MNGCs in *Esr1*-deficient ovaries were characterized by iron accumulation and strong autofluorescence. Finally, deletion of *Esr1* led to a significant increase in ovarian mast cells, involved in iron-mediated foam cell formation. Given that these findings are characteristics of ovarian aging, our data suggest that *Esr1* deficiency triggers mechanisms similar to those associated with aging.

## 1 Introduction

Estrogens, especially 17β-Estradiol (E2), play an essential role in a variety of biological processes within the female reproductive system. They control growth and differentiation of uterine tissue and successful ovulation (1). E2 exerts its functions by binding to specific estrogen receptors in the cytoplasm, which are subsequently translocated to the nucleus and initiate signaling via binding to estrogen response elements (2, 3).

The rodent ovary, which is an important E2 producing organ, expresses all three known estrogen receptors (3). However, their expression differs between functional compartments of the ovary. While estrogen receptor beta (ERβ, *Esr2*) is strongly expressed by granulosa cells in growing follicles (4–7), estrogen receptor alpha (ERα, *Esr1*) is more diffusely expressed in interstitial and thecal cells (6–8). It is therefore not surprising that misregulation or depletion of these receptors has severe effects on physiological processes and impairs fertility (9). The lack of these receptors leads in each case to quite different phenotypic characteristics (3, 10–13). The most severe phenotype occurs when *Esr1* is disrupted, resulting in infertility in both female and male mice (14), while depletion of *Esr2* is associated with sub-fertility and comparatively normal gross morphology (9, 15). The third receptor through which E2 can signal is G-protein coupled estrogen receptor 1 (GPER1). Interestingly, although GPER1 is expressed in the ovary and uterus, *Gper1*-deficient mice do not exhibit any reproductive defects (16).

For about 30 years, mice deficient in *Esr1* have been to study the molecular mechanisms responsible for infertility, and to link them to various ovarian diseases (9, 14, 17). Although *Esr1-*deficient females show follicular maturation up to the antral follicle stage, they do not ovulate (17). Therefore, no *corpora lutea* are formed in the ovaries of these animals, and instead follicular atresia or enlarged hemorrhagic cysts are formed (10, 17). A similar infertile phenotype was observed in mice lacking aromatase (*Cyp19*), the essential enzyme, which converts androgens to estrogens (18, 19). Recently, abnormal iron accumulation was reported in the ovarian stroma of young *Cyp19*-depleted females, indicating impaired iron homeostasis in context of endocrine disruption (20). Interestingly, physiological aging of the ovaries, which is associated with natural decline in fertility is also characterized by elevated iron levels (21–23). Similar findings have been noted in women with infertility-associated endocrine ovarian disorders such as polycystic ovary syndrome (PCOS) or endometriosis (24–26).

Iron is an important element that the body needs for a variety of processes, including physiological functions in female reproductive tract (27). An imbalance in iron homeostasis leads to tissue iron overload that in turn is capable to catalyze redox reactions, resulting in accumulation of toxic lipid peroxides that negatively affect folliculogenesis (28, 29). In general, iron can be present as ferrous (Fe^2+^) or in the oxidized ferric (Fe^3+^) state. Ferrous iron is mainly produced transiently because of its higher cellular toxcity, whereas ferric iron is the stable form (30). In the cell, trivalent iron is mainly transported by transferrin and stored in form of ferritin or hemosiderin (30). While ferritin is a physiological bioavailable intracellular storage protein, hemosiderin is an iron-storage aggregate consisting of partially degenerated ferritin and lysosomes, that are often formed after bleeding (31, 32). Hemosiderin accumulation has been observed in different tissues and within the ovaries where it is associated with natural aging, nulliparity and endocrine disease (21, 22, 33, 34). However, the exact function of hemosiderin and the consequences of its deposition in various body systems have not yet been conclusively clarified.

The balance of iron metabolism is ensured by a complex network of cells and proteins involved in transport, export, and import (30). In general, macrophages are described as important players in regulation of iron metabolism through their involvement in import/export, recycling and storage of iron (35). Ovarian macrophages show high levels of heterogenicity, having diverse functions in health and disease (36, 37). In naturally aging murine ovaries, these phagocytic cells are associated with iron accumulation (21, 23). In addition, aging triggers the differentiation of residential ovarian macrophages, leading to increased M2 polarization of macrophages (anti-infammatory) and the appearance of so-called marcophage-derived multinucleated giant cells (MNGCs) (22, 34, 38). Whether endocrine disorders such as the depletion of *Esr1* have similar effects on the ovary has only been speculated (34).

Recently, we reported that *Esr1*-deficient female mice show dramatically increased levels of Lipocalin 2 (LCN2) in ovarian tissue (8). LCN2, originally described as a neutrophil gelatinase-associated lipocalin, is a 25-kDa protein glycoprotein with diverse immunological and metabolic functions including iron transport (39–41). Although LCN2 cannot bind iron directly, it has the ability to chelate Fe^3+^ bound to siderophores. LCN2 uses bacterial siderophores as cofactors to form a complex, thereby limiting bacterial growth and infections by sequestering iron (42, 43). In addition, mammalian siderophores have been discovered that are potential ligands for LCN2 (44). Therefore, we speculate that the strong ovarian LCN2 expression in *Esr1*-deficient females (8) indicates altered iron homeostasis in these animals. To the best of our knowledge, there are currently no studies on whether or in what way iron homeostasis is affected by depletion of *Esr1* in the ovary. In this scenario, a change in iron metabolism is conceivable because an association between a hemorrhagic or cystic phenotype and iron excess has been noted in other ovarian disorders such as PCOS or endometriosis. Although these disorders are formed by different pathomechanisms, they are also related to infertility (24, 45–47). Similarly, natural aging of the ovaries is accompanied by a steady decline in fertility and impaired iron metabolism (21–23).

The aim of the present study was to investigate the consequences of depletion of *Esr1* on iron homeostasis. Therefore, we analyzed the ovaries of adult *Esr1*-deficient animals and aged-matched wild type animals with respect to iron regulation. In addition, we are speculating about the potential link between the depletion of *Esr1* and the early indicatiors of ovarian aging. The results of the present study highlight that endocrine dysfunction is associated with common signs of ovarian aging, such as significant accumulation of iron, the influx of macrophages and the presence of MNGCs.

## 2 Material and methods

### 2.1 Animal housing and tissue collection

Homozygote female and male *Esr1*-deficient mice are infertile (48). Therefore, *Esr1^–/–^* animals were obtained by mating heterozygote *Esr1* ^+/–^ (B6N(Cg)-*Esr1* ^tm4.2Ksk^/J) males and females, purchased from The Jackson Laboratory (JAX stock #026176, The Jackson Laboratory, Bar Harbor, ME, USA). The handling of the animals was carried out in accordance with the German animal welfare law (Tierschutzgesetz, TSchG) and the Directive 2010/63/EU. All experiments involving animal sacrifice and subsequent tissue dissection were approved by the internal Review Board of the RWTH University Hospital Aachen (permit no.: TV40138). Animal housing and care was carried out as previously described in (8), with mice maintained in a 12 h light/12 h dark cycle. The mice used in this study were between 12- and 22-weeks-old (or reproductively old between 77 and 79 weeks-old, cf. Section 3.5) at sacrifice that was carried out by cervical dislocation with prior isoflurane sedation. The freshly removed female reproductive organs (as well as liver and spleen tissues) were rinsed in phosphate-buffered saline (PBS) and then either fixed for histological analysis (24 h, 4°C in 4% neutral buffered formaldehyde, stabilized with methanol), or snap-frozen in liquid nitrogen and then stored at -80°C for further processing. Since the aim of this study was to compare the female reproductive tract of *Esr1*-deficient animals with that of wild type mice, only female animals were used. In the present study, we included wild-type females in all estrus phases to eliminate the possibility that the reported results are simply due to hormonal fluctuations. The corresponding males were used in the previous study (8), which provides additional information such as the specific genotyping protocol and analysis of the animals total body weights.

### 2.2 Histological analysis of tissue sections

For all histological procedures described in the following section, the tissue was treated equally. First, the tissue was fixed for 24 h, dehydrated, and then embedded in paraffin. The formaldehyde-fixated paraffin-embedded tissue blocks were stored at room temperature (RT) until sectioning. From the tissue, 3-µm thick sections were prepared and deparaffinized in xylene and subjected for rehydration to decreasing graded ethanol. In order to compare the different histological stains, consecutive sections were made. Unless otherwise stated, all further steps were performed at RT.

#### 2.2.1 Hematoxylin and eosin stain

The tissues were stained for 5 min in Mayers Hematoxylin (Liliess Modification, #S3309, Agilent Technologies, Inc., Santa Clara, CA, USA) diluted 1:3 in dH_2_O. Afterwards the tissues were washed under running tap water for 10 min. Cytoplasmic staining was done by placing the slides in Eosin pH 4.5 (#HT110216, Sigma-Aldrich, Taufkirchen, Germany) for 15 sec. The samples were dehydrated (in increasingly graded ethanol and xylene) and subsequently mounted in DPX mounting medium (#06522, Sigma-Aldrich). Hematoxylin and eosin (HE) staining was used for histological analysis of tissue structure and to determine the estrous phase of the wild type females. Details of the assessment and specific histological characteristics can be found in our previous study (8).

#### 2.2.2 Perls Prussian Blue staining

The Perls Prussian Blue (PPB) method, known as Berlin Blue Reaction, is a histochemical method for the detection of trivalent iron associated with hemosiderin in tissue sections (49). To identify iron deposits in reproductive tracts of *Esr1*-deficient and wild type females, Prussian Blue [Iron (III) Detection] staining kit (#11097, MORPHISTO, Offenbach am Main, Germany) was used according to manufacturers instructions. Briefly, after rehydration (see Section 2.2), the tissue samples were incubated for 5 min at 40°C in 5% potassium ferrocyanide (II) and subsequently in freshly prepared working solution of 5% potassium ferrocyanide (II) in hydrochloric acid solution (1:1) for 30 min at 40°C. After washing the section for 5 min in dH_2_O, counterstaining for 10 min with 0.1% Seed red (nuclear red) was performed. The tissue samples were dehydrated and mounted with DPX as previously described (see Section 2.2.1). The reaction product of the ferric iron with the potassium hexacyanoferrate (II) in hydrochloric acid solution precipitates as an insoluble blue complex salt, which is clearly distinguishable from pale pink stained tissue structures (49).

#### 2.2.3 Immunohistochemical detection with Perls Prussian Blue staining

To investigate whether the iron deposits co-localize with lipocalin 2 (LCN2), PPB was performed combined with immunohistochemical staining for LCN2. Therefore, the tissue sections were first treated as described in Section 2.2. Afterwards, antigen retrieval was done by heating the slices in sodium citrate buffer (10 mM, 0.05% Tween 20, pH 6.0) in a steamer for 30 min, followed by cooling on ice for 20 min). Next, the samples were washed in PBS and PBS supplemented with 0.1% Tween^®^ 20 (PBS-T). Then, Avidin/Biotin Blocking Kit (#SP-2001, Vector Laboratories, Newark, CA, USA) was used essentially as described in the manufacturers instructions. To block non-specific antibody binding sites, tissue sections were incubated in 5% normal rabbit serum (#X0902, Agilent Technologies) in blocking solution (1% BSA, 0.1% cold fish gelatin, 0.1% Triton-X-100, 0.05% Tween^®^ 20 in PBS) for 90 min. Primary anti-LCN2 Antibody (#AF3508, R&D Systems, Minneapolis, MN, USA) was diluted 1:40 in blocking solution and incubated on slides at 4°C overnight. Normal goat IgG (#AB-108-C, R&D Systems) were used at the same concentration as the primary antibody and served as a negative control. Next day, endogenous peroxidase was blocked by incubating the tissue slices in 3% hydrogen peroxide (#31642, Sigma-Aldrich) in dH_2_O. Tissue was then washed in dH_2_O and PBS-T and incubated with a biotinylated rabbit polyclonal anti-goat secondary antibody (#E0466, Agilent Technologies, diluted 1:300 in PBS), for 1 h. After washing in PBS-T, the slices were incubated in ABC-Complex solution (#PK-6100, VECTASTAIN^®^ Elite^®^ ABC-HRP-Kit, Vector Laboratories) according to manufacturers instructions for 1 h in RT. The chromogen 3,3-diaminobenzidine tetrahydrochloride (DAB, SIGMAFAST, #D9292, Sigma-Aldrich) was used to visualize LCN2 expression. Subsequently, the PPB staining kit was used as described above (Section 2.2.2). In brief, tissue sections were first incubated for 5 min at 40°C in 5% potassium ferrocyanide (II) and then for 30 min at 40°C in freshly prepared working solution of 5% potassium ferrocyanide (II) in hydrochloric acid solution (1:1). Tissue sections were washed for 5 min in dH_2_O and counterstained in 0.1% Seed red (nuclear red) for 5 min. Finally, samples were dehydrated and mounted with DPX as previously described (see Section 2.2.1).

#### 2.2.4 Periodic Acid-Schiff reaction

The Periodic Acid-Schiff (PAS) reaction is a histochemical staining technique to detect carbohydrate-containing components like glycogen which are typical for macrophages (50). To visualize polysaccharide-enriched phagocytic cells in the female reproductive tract, we used a PAS reaction staining kit (#12153, MORPHISTO) according to manufacturers instructions. In brief, after the rehydration steps (see Section 2.2), tissue sections were incubated in 1% periodic acid for 20 min, washed with H_2_O, and the Schiffs reagent was applied on the sections for 10 min. Finally, the tissue was counterstained with hematoxylin for 2.5 min, followed by ‘blueing under running tap water for 3 min. The tissue sections were dehydrated and mounted in DPX mounting medium as previously described (see Section 2.2.1).

#### 2.2.5 Toluidine blue staining

Toluidine blue (TB) is a dye for metachromatic staining of mast cells in tissues, which stains mast cell granules in purple and the background in blue (51). A standard protocol was used with a 1% Toluidine blue (#89640-25G, Sigma-Aldrich) stock solution in 70% ethanol. In brief, after the rehydration steps (see Section 2.2), sections were stained with 0.1% toluidine blue working solution in 1% sodium chloride for 5 min. After several immersions in distilled H_2_O, the tissue sections were dehydrated in ethanol and xylene and mounted in DPX as previously described (see Section 2.2.1). The number of mast cells per mm^2^ was determined by counting the positive (purple stained) mast cells in the ovary. For this purpose, the stained tissue sections were scanned and NDP.view2 software (see Section 2.2.8) was used to determine the areas of the ovaries.

#### 2.2.6 Detection of autofluorescence

Autofluorescence of tissues may be due to the accumulation of lipofuscin, an aging pigment that is stored in phagocytic cells (22, 23). For visualization of autofluorescence in reproductive tissue section, slices were deparaffinized, rehydrated and embedded in aqueous PermaFluor™ mounting medium (#TA-030-FM, Thermo Fisher Scientific Inc., Waltham, MA, USA) with or without nuclear counterstaining in 4,6-diamidino-2-phenylindole dihydrochloride (DAPI) solution (#D1306, Thermo Fisher Scientific).

#### 2.2.7 Immunofluorescence staining

Formalin-fixed, paraffin-embedded tissue sections with a thickness of 3 µm were used for immunofluorescence staining and were deparaffinized and prepared as described above (see Section 2.2). Subsequently, heat-induced antigen retrieval was done in a steamer by placing the slides in citrate buffer (10 mM, pH 6.0, 0.05% Tween^®^ 20) for 30 min, followed by cooling on ice for 20 min. Next, samples were rinsed with PBS and PBS-T. Non-specific binding sites were blocked with 5% normal donkey serum (#ab7475, Abcam, Cambridge, UK) in PBS supplemented with blocking solution (0.1% cold fish skin gelatine, 1% bovine serum albumin, 0.1% Triton X-100, and 0.05% Tween^®^ 20) for 90 min. Tissue sections were incubated with primary antibodies against F4/80 (1:50, #MCA497G, Bio-Rad Laboratories GmbH, Dusseldorf, Germany) and LCN2 (1:40, #AF3508 R&D Systems) or with IgG controls (normal rat IgG_2b_,#65211-1-Ig, Proteintech, Planegg-Martinsried, Germany; normal goat IgG, #AB-108-C, R&D Systems) in blocking buffer overnight at 4°C. IgG controls were used at the same concentration as the primary antibodies. All the following steps were carried out under exclusion of light. The next day, the sections were washed and incubated simultaneously with two fluorescently-labeled secondary antibodies (donkey anti-goat Alexa Fluor Plus 555, #A32816, Thermo Fisher Scientific and donkey anti-rat Alexa Fluor Plus 488, #A11208, Thermo Fisher Scientific), diluted 1:300 in PBS for 1 h. The TrueBlack^®^ Lipofuscin autofluorescence quencher solution (#23007, Biotium, Fremont, CA, USA) was used to quench autofluorescence in reproductive tissue samples (52). For this purpose, the sections were incubated for 3 min in the TrueBlack^®^ solution freshly diluted (1:20) in 70% ethanol. Thereafter, the tissue sections were washed with PBS and nuclear counterstaining was performed for 30 min using a 200 ng/ml DAPI solution in PBS. Finally, tissue slices were mounted with aqueous PermaFluor™ mounting medium and stored in the dark at 4°C until fluorescence microscopic evaluation.

#### 2.2.8 Imaging

Images were taken with a Nikon Eclipse E80i fluorescence microscope, equipped with the NIS-element Vis software (Version 3.22.01, Nikon, Tokio, Japan). In addition, selected tissue slides were scanned using a NanoZoomer (#C13220-04, Hamamatsu, Naka-ku, Japan), viewed using NDP.view2 software (version U12388-01, Hamamatsu), and exported to create final images.

### 2.3 Laser Ablation Inductively Coupled Plasma Mass Spectrometry (LA-ICP-MS)

Ovarian tissues of female animals (Wild type: n=5, *Esr1^–/–^*: n=5) aged 12-15 weeks were used for laser ablation inductively coupled plasma mass spectrometry (LA-ICP MS) measurement.

#### 2.3.1 Tissue preparation for LA-ICP-MS

Protocols for standardized quantitative LA-ICP-MS analysis with liver tissue were previously established by us (53). In these protocols, it is necessary to flush the tissue prior measurement with saline buffer for detection of proper endogenous iron concentrations. In unflushed liver, the majority of the measured iron concentration results from the blood and thus can lead to massive misinterpretations (54). Therefore, sacrificed animals were immediately transcardially perfused. For this purpose, a 26G needle was inserted from the tip of the heart about 5 mm into the left ventricle and fixed by means of hemostatic forceps (‘Mathieu needle holder). Then a small incision was made in the right atrium with fine scissors and the perfusion with sterile PBS was carefully started and continued until the liver turned pale. Afterwards the tissues were carefully dissected and frozen at -80°C. For preparing slides for LA-ICP-MS analysis, the samples were cut into 30 µm thick slices using a cryomicrotome (#CM3050S, Leica Biosystems, Wetzlar, Germany), in which the temperature of the cryo-chamber was set to -27°C and the object area temperature to -24°C. The slices were mounted on StarFrost^®^ self-adhesive microscope slides (B4 0303, Knittel Glass, Braunschweig, Germany) and stored at -80°C until analysis. Before the sections were subjected to LA-ICP-MS measurement, the cryosections were scanned with a slide scanner (see Section 2.2.8) to obtain a light microscopic overview. Microscopic images were viewed and analyzed using the NDP.view2 software.

#### 2.3.2 LA-ICP-MS measurement and analysis

The LA-ICP-MS technology consists of a line-by-line ablation of tissue material with a fine, focused laser beam. The ablated tissue is then transferred into the inductively coupled plasma source of a mass spectrometer using an inert carrier gas stream (*e.g*., argon). After being vaporized, atomized and ionized, the molecules are split according to its mass-to-charge ratio (55, 56). In our study, trace element measurements were done in a quadrupole-based inductively coupled plasma mass spectrometer (8900 ICP-MS, Agilent Technologies) that was linked to a laser ablation system (New Wave NWR213; Elemental Scientific, Omaha, NE, USA). In addition to ^56^Fe, the following isotopes were routinely monitored and quantified as part of the established LA-ICP-MS analysis: ^13^C, ^23^Na, ^24^Mg, ^31^P, ^34^M, ^39^K, ^44^Cr, ^55^Mn, ^63^Cu and ^64^Zn. Generation, analysis and visualization of LA-ICP-MS data to obtain metal distribution in the tissues was done with the Excel-based Laser-Ablation Imaging (ELAI) program, consisting of Microsoft Excel with Visual Basic for Application (VBA), as described elsewhere (57, 58). For the determination of the elemental concentrations in µg/g tissue, standards including different well-defined concentrations of each element have been prepared from homogenized liver tissues. The concentration of each isotope was normalized to the mean intensity of ^13^C ion intensity per tissue as an alternative marker for sample thickness. More detailed information regarding experimental set up during LA-ICP-MS measurement, calibration and standard preparation are described elsewhere (53, 56, 57, 59, 60).

### 2.4 RNA analysis

Parts of snap-frozen female tissue were placed in RNA lysis buffer with DTT and homogenized as described previously (4). Protocols for RNA extraction and purification including DNase digestion followed by reverse transcription and quantitative real-time PCR (RT-qPCR), and evaluation were previously published (8). A list of all primers used in this study is given in **Supplementary Table 1**.

### 2.5 Protein analysis by Western blot

Female tissues were placed in RIPA buffer (on ice) containing 20 mM Tris-HCl (pH 7.2), 150 nM NaCl, 2% (*w*/*v*) NP-40, 0.1% (*w*/*v*) SDS, and 0.5% (*w*/*v*) sodium deoxycholate supplemented with the Complete™ mixture of phosphatase inhibitors (#P5726-1ML, Sigma-Aldrich). For ovarian protein analysis, both ovaries of each female were pooled. Tissue homogenization was performed with a mixer mill and protein extraction and Western blot analysis was performed as described elsewhere (8). 70 µg protein samples per lane were loaded for liver and spleen tissue analysis and 60 µg for ovaries. An overview of the antibodies used in this study is given in **Supplementary Table 2**.

### 2.6 Data analysis

All calculations were done in Excel v16 (Microsoft Corporation, Redmond, WA, USA). Statistical analysis was performed with GraphPad Prism v.8.0 (GraphPad Software, Inc., La Jolla, CA). Gaussian distribution was tested with Shapiro-Wilk Tests. Subsequently, Students *t*-test was used if normality could be assumed, while otherwise a non-parametric Mann-Whitney Test was applied. All data in this study is shown as mean ± standard deviation (SD). Statistical significance between groups was assumed when probability values were below 0.05 (*p* < 0.05). Significant differences are indicated by asterisks: * *p* < 0.05, ** *p* < 0.01, *** *p* < 0.001.

## 3 Results

### 3.1 LA-ICP-MS analysis of murine ovaries

In reproductive organs, especially in the ovary, studies have shown that unbalanced iron homeostasis, is associated with unexplained infertility (61, 62). Therefore, we speculated that depletion of *Esr1*, which leads to infertility, may be associated with imbalance in iron homeostasis. To test our hypothesis, we used the LA-ICP-MS technology that represents a powerful imaging technique that can be used to determine the distribution of iron (isotopes) in biological tissue in health and disease (55, 57).

**Figure 1:**
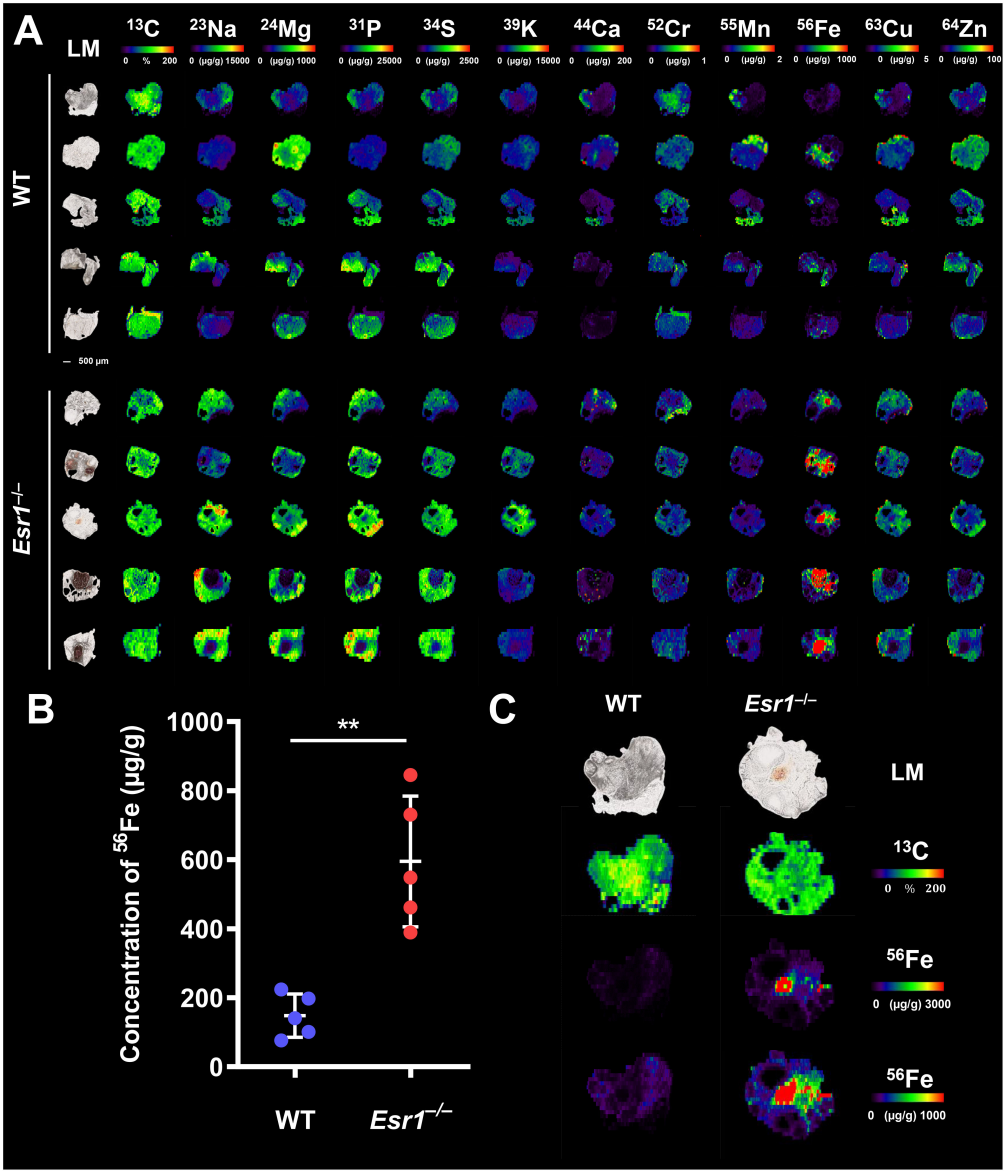
Laser ablation inductively coupled plasma mass spectrometry (LA-ICP-MS) imaging of wild type (WT) and *Esr1*-deficient ovaries. LA-ICP-MS imaging was performed on 30 µm-thick cryosections and the distribution of different isotopes was determined. Details are described in the Material and Methods section. **(A)** Overview of all analyzed isotopes in ovaries of WT (n=5) and *Esr1*-deficient (n=5) animals. Individual images were generated with the ELAI software tool. Light microscopy (LM) images of the cryosections of the individual ovaries are shown on the left side. Scale bar represents 500 µm. The content of ^13^C used for normalization is presented in %, while other isotope concentrations are shown in μg/g tissue. **(B)** The concentration of ^56^Fe (in µg/g) is significantly higher in *Esr1*-deficient ovaries compared to WT controls. For statistical analysis a Students *t*-test was performed. The significant difference between both groups is indicated by asterisks: **p<0.01. **(C)** Images of ^56^Fe distribution are shown at different concentration-based scales for a representative WT and *Esr1*-deficient ovary. The ^56^Fe isotope concentration-based scale was lowered from 0-3,000 µg/g to 0-1,000 µg/g to highlight ^56^Fe accumulation in *Esr1*-deficient animals.

To determine whether the absence of *Esr1* affects iron load in murine ovaries, we subjected cryosections of *Esr1*-deficient and wild type (WT) animals to LA-ICP-MS imaging. In total 12 different isotopes were measured (57, 58). The calculated concentrations of the individual isotopes refer in each case to the total ovarian tissue shown and are given in µg/g tissue (**Supplementary Table 3**). The LA-ICP-MS analysis of the ovarian tissue of *Esr1*-deficient and WT animals showed no differences in the majority of the studied isotopes. Only ^52^Cr (higher in WTs), ^23^Na and ^56^Fe (both increased in *Esr1*-deficient) showed significant differences between the two groups (**Figure 1A**; **Supplementary Table 3**). Interestingly, statistical analysis revealed significantly higher ^56^Fe iron content in *Esr1*-deficient ovaries (**Figure 1B**). These animals had approximately 4-fold higher iron concentration in the ovary (595 ± 189 µg/g) compared to WTs (148 ± 63 µg/g). The increased iron concentration (^56^Fe) is clearly evident when the concentration-based scale (usually 0-1,000 µg/g) was expanded to 0-3,000 µg/g (**Figure 1C**). Defined areas of high iron content in the *Esr1*-deficient ovary are marked by red color. The light microscopic analysis further revealed that increased iron content in *Esr1*-deficient animals is most likely localized to the ovarian stroma.

From the LA-ICP-MS analysis, we concluded that *Esr1*-deficient animals had significantly higher amount of iron in the ovaries compared to WT animals. However, these measurements do not allow the determination of exact form of iron present, and therefore the iron metabolism of the animals was further investigated.

### 3.2 Analysis of different iron-regulators in *Esr1*-deficient ovaries and liver

**Figure 2:**
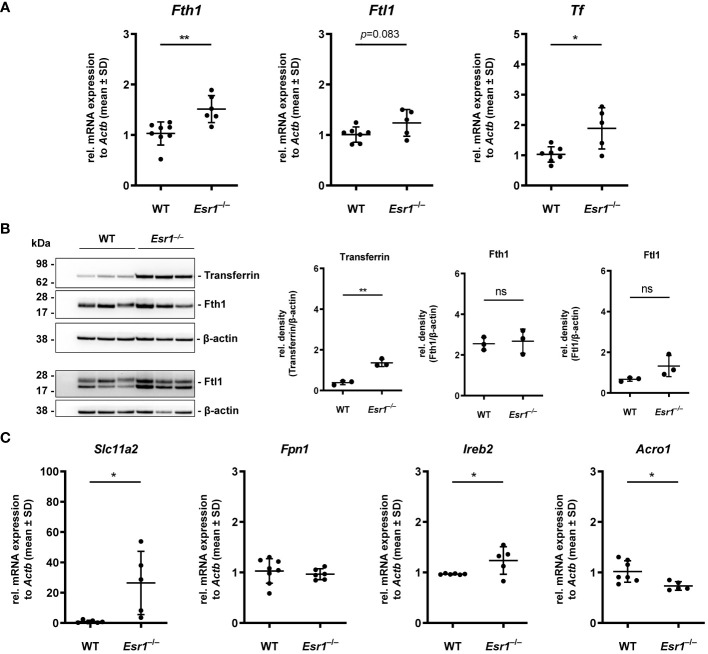
Analysis of key players in cellular iron metabolism handling in ovarian tissue. Wild type (WT) and *Esr1*-deficient ovarian tissues were either used for mRNA (WT, n=7; *Esr1* ^–/–^, n=5) or protein analysis (WT, n=3; *Esr1* ^–/–^, n=3). Relative mRNA expression of *Fth1*, *Ftl1* and *Tf* were measured by RT-qPCR and validated by **(B)** Western blot analysis. Protein expressions of Transferrin, Fth1 and Ftl1 were quantified densitometrically and plotted relative to β-actin expression. **(C)** mRNA expression of *Slc11a2*, *Fpn1*, *Ireb2* and *Aco1* were detected by RT-qPCR. All data **(A-C)** are displayed as mean ± SD. For statistical analysis a Students *t*-test was performed. Significant differences between groups are marked with asterisks: *p<0.05, **p<0.01, ns = not significant.

Considering the increase in iron in murine ovaries of *Esr1* ^–/–^ mice, we speculated that proteins and genes associated with iron metabolism might be altered in these animals. Therefore, we next investigated the expression of key mediators involved in cellular handling of iron, including iron storage (ferritin, transferrin), iron export/import (ferroportin, divalent metal transporter 1) and iron regulation (iron regulatory protein 1/2) proteins. Importantly, we detected significantly enhanced expression of ferritin heavy chain 1 (*Fth1)* and a trend to higher ferritin light chain (*Ftl1*) in *Esr1*-deficient ovaries at the mRNA level (**Figure 2A**). In addition, significantly increased expression of transferrin (*Tf*) was detected in these animals. In line, Western blot analysis revealed significantly higher ovarian transferrin protein in *Esr1*-depleted mice than in WT controls (**Figure 2B**). Furthermore, solute carrier family 11 member 2 (*Slc11a2*), a metal importer, was significantly enhanced in *Esr1-*deficient ovaries compared with WT animals (**Figure 2C**). However, there was no change in ferroportin (*Fpn1*) expression, which is an important iron exporter/efflux pump. Moreover, we found significantly higher expression of iron responsive element binding protein 2 (*Ireb2)*, but lower levels of aconitase *(Aco1)* in *Esr1*-deficient compared to WT ovaries.

Increased iron deposition and altered iron metabolism are signs of stress to which cells react by upregulating critical adaptive response mechanisms such as heme oxygenase 1 (HMOX1) (63). Interestingly, we detected significantly higher mRNA expression of *Hmox1* in *Esr1*-deficient ovaries compared to WT animals, which was reflected by a slight but not significant increase of HMOX1 protein (**Supplementary Figures 1A, B**). Iron overload can drive cellular death (known as ferroptosis), which is prevented by *e.g*., the glutathione peroxidase signaling (GPX4) pathway (64). However, no differences in *Gpx4* mRNA or protein expression was detected between WT and *Esr1*-deficient ovaries (**Supplementary Figures 1C, D**).

**Figure 3:**
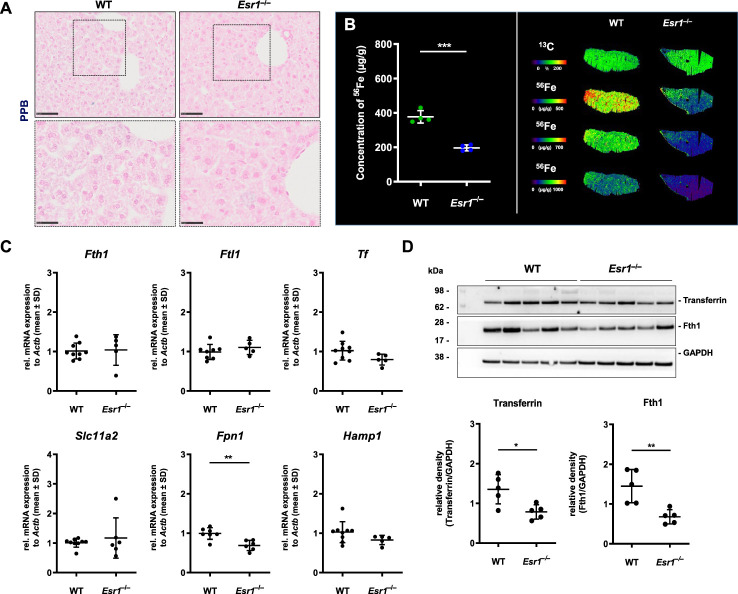
Analysis of cellular iron metabolism in liver tissue. Wild type (WT) and *Esr1*-deficient liver tissues were used for different analysis. **(A)** Formalin-fixated paraffin-embedded liver tissues sections (WT, n=4; *Esr1* ^–/–^, n=4) were stained for iron using Perls Prussian Blue (PPB). The scale bars equal to 50 µm (solid boarder) or 25 µm (dashed border). **(B)** Liver cryosections were subjected to LA-ICP-MS imaging. Concentration of elemental iron isotope (^56^Fe) (µg/g) is significantly lower in *Esr1*-deficient livers compared to WTs (left panel). ^56^Fe distribution is shown for both genotypes at different concentration-based scales for a representative WT and *Esr1*-deficient liver (left panel). **(C)** Regulators of cellular iron metabolism (*Fth1*, *Ftl1*, *Tf*, *Dmt1*, *Fpn1*, and *Hamp1*) were analyzed by RT-qPCR. **(D)** Protein expression of transferrin and Fth1 was investigated by Western blot analysis. Expression levels were quantified densitometrically and plotted relative to GAPDH expression. All data **(B–D)** are displayed as mean ± SD. For statistical analysis a Students *t*-test was done. Significant differences between groups are marked with asterisks: *p<0.05, **p<0.01, ***p<0.001.

Since our results indicated enhanced iron storage and transport in the *Esr1*-deficient ovary, the question arose about whether this is a local occurrence limited to ovaries or is also systemically reflected in these animals. To address this issue, we next analyzed the expression of iron in the liver as the site of iron synthesis and the spleen as an iron recycling organ (35). To do so, liver tissue slices from female WT and *Esr1* null mice were subjected to Perls Prussian blue (PPB) staining. Since PPB exclusively stains the non-heme iron in the cells, the ferric Fe^3+^-positive cells (stained blue) can be clearly distinguished in the staining from erythrocytes present in the blood (stained red) (49). This analysis revealed a very low overall positive staining for iron in parenchymal liver cells (**Figure 3A**). Several PPB-positive cells were detected in the *Esr1*-null livers, whereas positive cells were only rarely found in the WT livers (**Figure 3A**). In line, LA-ICP-MS imaging (**Figure 3B**) revealed that livers of WT animals have significantly higher amounts of isotope ^56^Fe (196.5 ± 17.6 µg/g) compared to *Esr1*-deficient animals (377.3 ± 36.11 µg/g). Western blot analysis of Fth1 and transferrin further showed significantly lower expression of these iron storage proteins in *Esr1*-deficient livers (**Figure 3C**). However, at mRNA level, there were no significant differences in hepatic expression of key players in iron metabolism (*Fth1, Ftl1, Tf, Slc11a2, Hamp1*) between WT and *Esr1*-deficent animals (**Figure 3D**). Only the expression of *Fpn1* was significantly decreased in *Esr1*-deficient livers.

To confirm that there was no systemic iron overload, splenic tissue of *Esr1*-deficent and WT mice were stained with PPB to visualize location of iron deposits. This analysis revealed iron-laden cells predominantly in the red pulp and occasionally in the white pulp of the spleen (**Supplementary Figure 2A**). Strikingly, *Esr1*-deficient animals showed no excess but even quite in opposite a lower amount of iron compared to the WTs. This corresponds to the similar Fth1 and transferrin expression in both groups as determined by Western blot analysis (**Supplementary Figure 2B**).

In conclusion, we found that different players of iron homeostasis are altered in *Esr1*-deficient ovaries, which are associated with iron overload conditions. Our data further indicate that there is no systematic iron overload in other organs involved in iron regulation (*i.e.*, liver and spleen).

### 3.3 Ovaries of *Esr1*-knockout mice show iron accumulation and LCN2 upregulation

Hemosiderin, an iron storage aggregate with poor accessibility, can be formed during iron overload when cells reach their capacity to bind iron to ferritin, such as during hemorrhages (65). This can be easily analyzed in routine hematoxylin and eosin (HE) staining in which hemosiderin appears as golden-brown aggregates (21).

**Figure 4:**
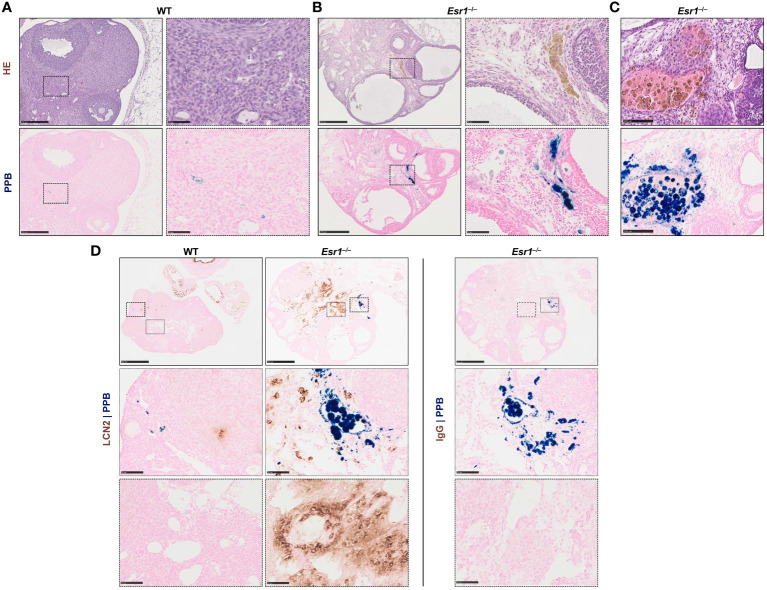
Hemosiderin deposits in ovarian tissues. Formalin-fixated paraffin-embedded ovarian tissues section of wild type (WT, n=8) and *Esr1*-deficient (n=8) animals were used for staining. Serial tissue slices of WT **(A)** and *Esr1*-deficient **(B)** ovaries were stained with Hematoxylin-Eosin (HE) or Perls Prussian Blue (PPB) to detect iron in form of hemosiderin. Hemosiderin can be seen in HE staining as brown-gold deposits and turns blue with PPB. The scale bars equal to 250 µm (solid boarder) or 50 µm (dashed border). Hemosiderin accumulations were found in ovarian stroma of *Esr1*-deficient animals but not in WTs and were localized around hemorrhagic cysts **(C)**. The scale bars equal to 100 µm. **(D)** Immunohistochemical localization of lipocalin 2 (LCN2) was combined with PPB staining to examine whether iron-positive cells co-localize with LCN2-positive cells. WT (n=3) and *Esr1*-deficient (n=4) ovaries were stained, showing no co-localization of iron and LCN2. Normal goat IgG was used as a negative control instead of the primary antibody. The scale bars equal to 500 µm (solid boarder) or 50 µm (dotted boarder and dashed border).

Since iron transport and storage is increased in *Esr1*-deficient ovaries (c.f. **Figure 2**), we next investigated whether hemosiderin is present in ovarian tissue. Therefore, ovarian tissue sections of adult *Esr1*-deficient and WT animals were initially stained with HE and serial sections were stained with Perls Prussian Blue (PPB) to confirm the ferric Fe^3+^ deposition (hemosiderin) (**Figures 4A, B**, *upper panels*). Interestingly, large amounts of golden-brown deposits were evident in the ovaries of *Esr1*-deficient animals throughout the tissue section, while in contrast no deposits were seen in the WT animals. These accumulations were preferentially located in the interstitial ovarian stroma (**Figure 4B**) or in close proximity to hemorrhagic cysts (**Figure 4C**, *upper panel*). PPB staining confirmed that the golden-brown deposits were hemosiderin accumulations (**Figures 4A, C**, *lower panels*). While the WT ovaries showed only sporadic, blue-stained cells, the ovaries of *Esr1*-deficient animals exhibited many dark, blue-stained areas that coincided with the deposits occurring in HE staining. In addition to the dark blue stains, the ovaries of *Esr1*-depleted mice also contained light-blue colored cell clusters (see Section 3.5).

In our previous study we demonstrated dramatically increased lipocalin-2 (LCN2) expression in female *Esr1*-deficient ovaries (8). Since this protein is an important player in iron homeostasis (44), we investigated in the following whether the same cells are responsible for both producing LCN2 and accumulating iron. Therefore, we combined immunohistochemical detection of LCN2 with subsequent PPB staining. Ovarian tissues of WT and *Esr1*-deficient animals were stained according to the described method (**Figure 4D**). In agreement with our previous report, WT ovaries contained only few LCN2-positive cells. In addition, we found no double-positive cells in WT ovaries. However, *Esr1*-deficient ovaries showed many cells that were positive for LCN2 and iron. Normal goat IgG that were used as a negative control showed no brownish stain that were observed after staining with the LCN2 specific antibody. Importantly, the results show that the vast majority of LCN2-positive cells do not coincide with the iron-loaded cells.

Taken together, staining with PPB revealed dramatically increased hemosiderin quantities in *Esr1*-deficient ovaries. The data further indicate that cells that are strongly positive for LCN2 are different than those in which iron is deposited. Nevertheless, it is conceivable that the high expression of LCN2 affects the iron deposition and metabolism. Possibly the iron is associated with phagocytic cell types and other immune cells, as it is the case in a different hemorrhagic disease model (66).

### 3.4 Macrophages in *Esr1*-deficient ovaries

**Figure 5:**
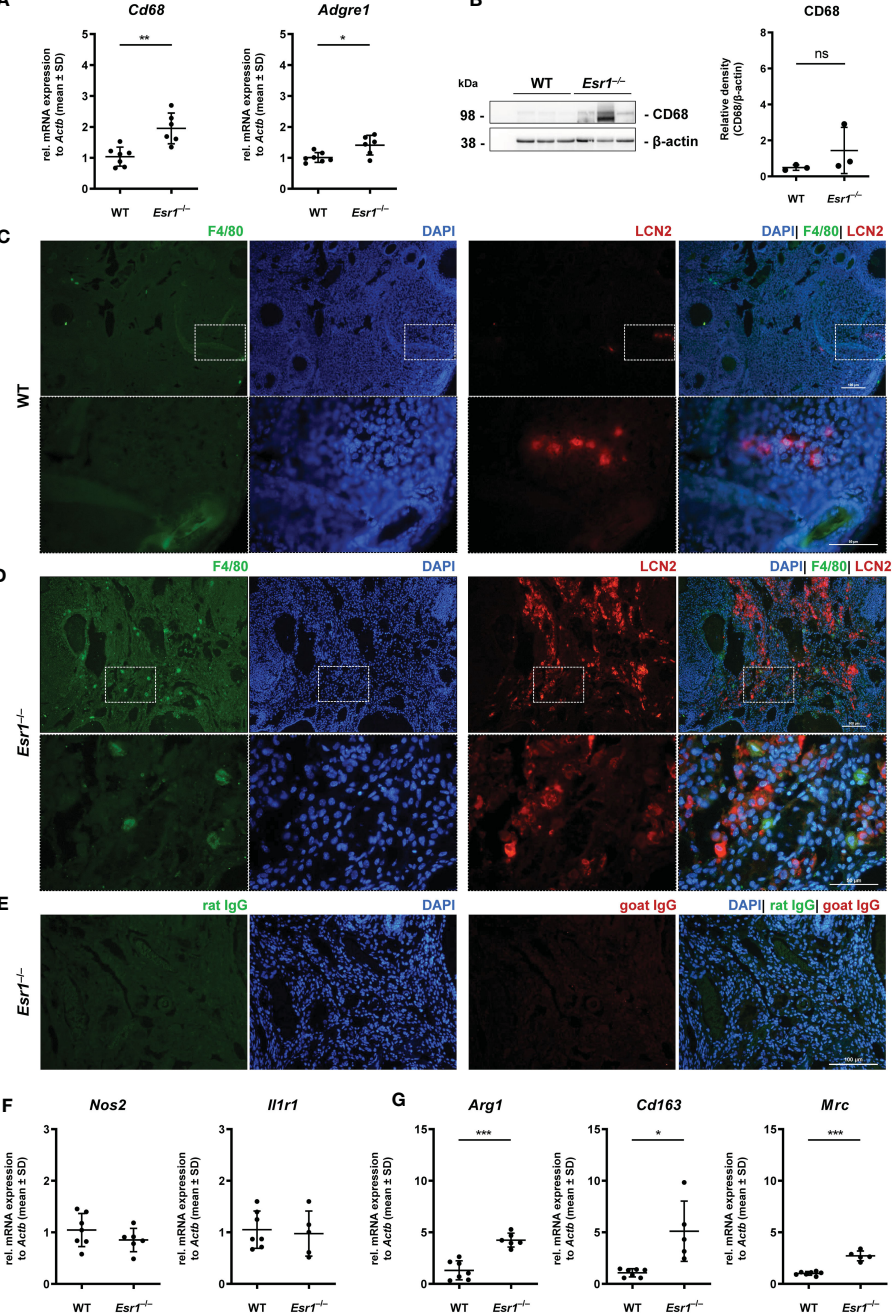
Macrophages in ovarian tissues. Wild type (WT) and *Esr1*-deficient ovarian tissues were either used for mRNA (WT, n=7; *Esr1* ^–/–^, n=5-6), protein analysis (WT, n=3; *Esr1* ^–/–^, n=3) or immunofluorescence staining (WT, n=3; *Esr1* ^–/–^, n=3). **(A)** Relative mRNA expression of pan-macrophage markers *Cd68* and *Adgre* were detected by RT-qPCR. **(B)** Western blot analysis was used to detect CD68 protein expression, which was quantified densitometrically and plotted relative to β-actin expression. F4/80 (green) and LCN2 (red) protein expression was visualized by immunofluorescence staining with nuclear DAPI counterstaining in **(C)** WT and *Esr1*-deficient **(D)** ovaries. **(E)** Normal rat and goat IgG were used as a negative control instead of the primary antibodies. The scale bars equal to 100 µm (solid boarder) or 50 µm (dashed border). **(F)** Relative mRNA expression of *Nos2* and *Il1r1*, as well as *Arg1*, *Cd163* and *Mrc* **(G)** were determined by RT-qPCR. All data **(A-B, F-G)** are displayed as mean ± SD. For statistical analysis, Students *t*-test was done. Significant differences between groups are marked with asterisks: *p<0.05, **p<0.01, ***p<0.001, ns =not significant.

Ovarian macrophages are an essential cell type for ovarian iron metabolism (37). Therefore, we hypothesize that the severe iron accumulation in the *Esr1*-deficient animals is linked to macrophage influx in these tissue. To test this hypothesis, we first performed RT-qPCR to investigate whether WT and *Esr1*-deficient animals differ in terms of ovarian macrophage expression. We found significantly higher expression of pan-macrophage markers *Cd68* (known as well as macrosialin) and *Adgre1* (encoding F4/80 protein) in *Esr1*-depleted ovaries when compared to WTs (**Figure 5A**). Moreover, Western blot analysis showed a clear trend of higher CD68 protein expression in *Esr1*-deficient animals (**Figure 5B**). The mRNA analysis was in line with results from immunohistochemical detection of F4/80 in the ovaries showing no- to low-numbers of F4/80-positive macrophages in the WT ovaries (**Figure 5C**). In WT ovaries, these cells were found in atretic follicles and regressing *corpus luteum*. Interestingly, the number of F4/80-positive cells were increased in ovaries of *Esr1*-deficient animals, which were located in the ovarian stroma (**Figure 5D**). Co-staining with LCN2 demonstrated no co-localization of F4/80-positive macrophages with LCN2-positive cells in either WT or *Esr1*-deficient ovaries (**Figures 5C, D**). As negative controls for immunofluorescence staining normal rat and goat IgGs instead of primary antibodies have been used, showing only a low background staining (**Figure 5E**). Furthermore, we investigated whether the increase in macrophages was predominantly due to M1-like (pro-inflammatory) or M2-like (anti-inflammatory) macrophages. Based on our previous findings of significantly elevated *Tnf* expression in *Esr1*-deficient ovaries, we anticipated an increase in pro-inflammatory macrophages. However, we observed no disparity in *Nos2* or *Il1r1* mRNA expression between WT and *Esr1*-deficient ovaries, which commonly used markers for M1-like macrophages (**Figure 5F**). In sharp contrast, there was a significant increase in all analyzed M2-like macrophage markers (*Arg1, Cd163, Mrc*) in *Esr1*-deficient compared with WT ovaries (**Figure 5G**).

Overall, our results demonstrate a strong increase in phagocytic cells in the *Esr1*-deficient ovary. Interestingly, we found significantly higher expressions of different M2-like macrophage markers in these animals, most likely indicating enhanced remodeling and tissue repair activity.

### 3.5 *Esr1*-deficient ovaries exhibit multinucleated giant cells

**Figure 6:**
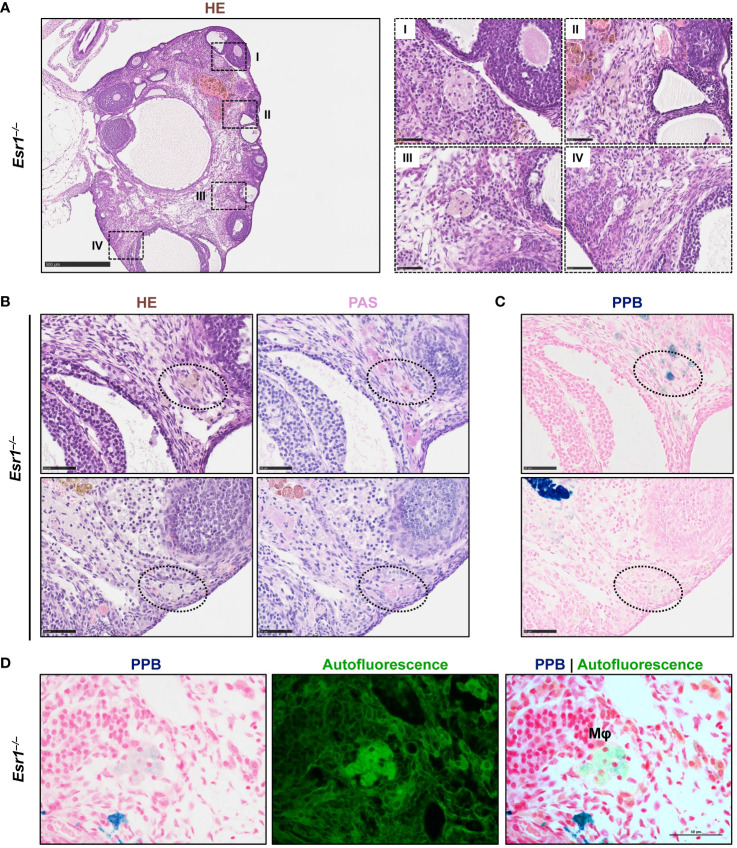
Multinucleated giant cells (MNGCs) are present in ovarian stroma of *Esr1*-deficient mice. Formaldehyde-fixated, paraffin-embedded serial ovarian tissue sections from *Esr1*-deficient mice (n=7) were used for different histological stainings. **(A)** Hematoxylin-Eosin (HE) staining shows pale cell clusters with a foamy appearance and multiple nuclei (I-IV) throughout the section of the *Esr1*-deficient ovary. **(B)** Cell clusters were identified as MNGCs by positive Periodic Acid-Schiff (PAS) reaction. **(C)** Perls Prussian Blue (PPB) staining revealed iron inclusions in the MNGCs. **(D)** PPB-stained MNGCs show strong autofluorescence. Scale bar equals 500 µm (solid boarder in **(A)**) or 50 µm (dashed boarder in **(A)** and in **(B–D)**). Mφ, macrophage.

In the last decade, a unique ovarian macrophage-derived multinucleated giant cell population (termed as multinucleated giant cell, MNGC) has been exclusively found in aged murine ovaries (22, 23, 34), but speculated to be affected by altered endocrine processes. Therefore, we next examined *Esr1*-deficient and WT ovaries for the presence of MNGCs. Interestingly, in HE staining, we detected several clusters of pale brownish cells in ovaries of *Esr1*-depleted mice (**Figure 6A**), which most likely represent MNGCs. Higher magnification of these clusters showed that the cells appeared foamy and contained multiple nuclei (**Figure 6A**, I-IV). By phagocytosis, MNGCs engulf and store diverse products, including polysaccharides, which can be positively stained by Periodic Acid Schiff (PAS) reaction (22, 50). To verify these characteristic, serial sections of ovarian tissue from *Esr1-*deficient animals were stained with HE and PAS, revealing that the MNGC clusters which appeared pale brownish in HE (**Figure 6B**, *left panels*) were strongly positive (stained pinkish) in PAS reaction **Figure 6B**, *right panels*). In addition, we could confirm that these MNGCs contained iron, as they appeared colored light blue in PPB staining (**Figure 6C**). Another characteristic of MNGCs is their strong autofluorescence compared with the surrounding ovarian tissue (22). Observation of the PPB-stained *Esr1*-deficient ovary slices under a fluorescence microscope revealed that foamy ovarian MNGCs exhibited strong autofluorescence (**Figure 6D**). Finally, we found that MNGCs are present in aged ovaries, as we detected small numbers of these cells in old animals of all groups (WT, *Esr1* ^–/–^ and *Esr1* ^+/–^). These cell clusters are visible in HE staining, are PAS reaction positive, contain iron accumulations, and exhibit strong autofluorescence (**Supplementary Figures 3A–D**).

In conclusion, we detected several clusters of MNGCs in the ovaries of 3-month-old *Esr1*-deficient mice, but not age-matched WT controls. These findings indicate that disruption of *Esr1* leads to signs of reproductive aging, confirming altered endocrinology as an underlying cause of MNGCs formation.

### 3.6 *Esr1*-deficient ovaries show high number of mast cells

**Figure 7:**
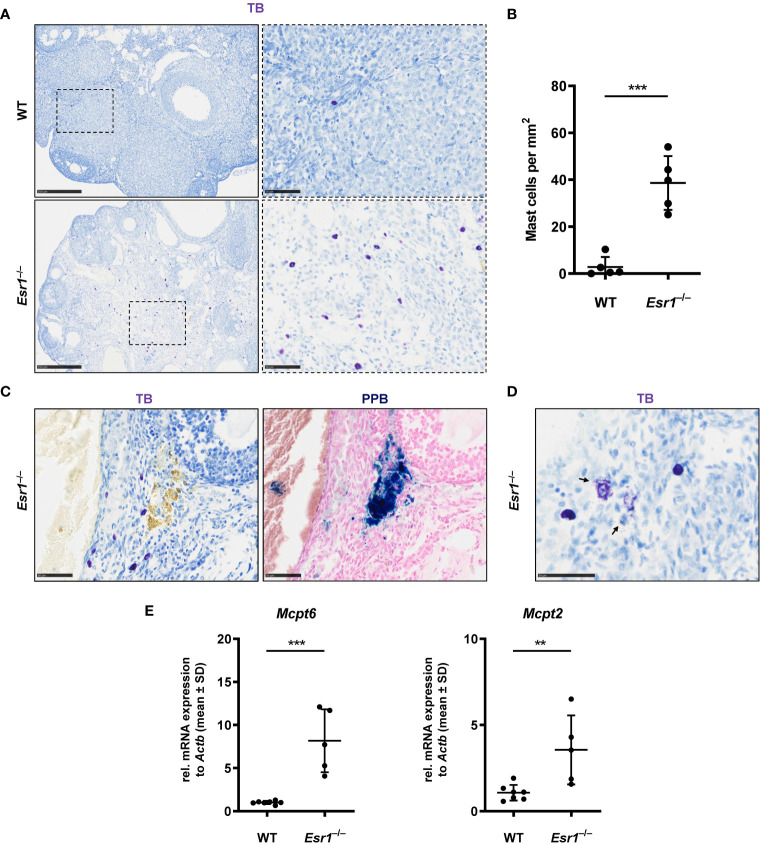
Increased number of mast cells in *Esr1*-deficient ovaries. Ovaries from wild type (WT, n=5) and *Esr1*-deficient mice (*Esr1* ^–/–^, n=5) were dissected and formalin-fixed paraffin-embedded tissue section were prepared. Toluidine blue (TB) staining was performed on ovarian tissue sections to detect mast cells. **(A)** WT animals demonstrate lower number of (purple stained) mast cells in the ovary than *Esr1* ^–/–^ animals. Scale bar correspond to 250 µm (solid boarder) or 50 µm (dashed boarder). **(B)** Evaluation of all female mice shows that *Esr1* ^–/–^ animals have significantly more mast cells per mm^2^ in the ovary compared to WT controls. Each dot represents an individual mouse and horizontal lines indicate the mean (± SD). **(C)** Serial sections either stained with TB or Perls Prussian Blue (PPB) demonstrate that some mast cells in *Esr1* ^–/–^ animals reside in the vicinity of iron-accumulated areas (blue precipitates). Scale bar corresponds to 50 µm. **(D)** Occasionally, degranulated mast cells were found in the *Esr1* ^–/–^ ovary. Arrows indicate release of mast cell granules into surrounding ovarian tissue. Scale bar equals 50 µm. **(E)** RT-qPCR was performed to evaluate relative mRNA levels of mast cell specific markers (*Mcpt6*, *Mcpt2*). **(B, D)** Students *t*-test was used for statistical analysis. Significant differences between groups are marked with asterisks: **p<0.01,***p<0.001.

Mast cells are present in the ovary at all stages of rodent estrous cycle and human ovaries and can release various mediators such as tumor necrosis factor alpha (13, 67–70). In addition, mast cells are involved in iron-induced conversion of macrophages to foam cells during hemorrhage (66). In the last part of this study, we determined the number of mast cells in ovaries of female WT and *Esr1*-deficient mice by toluidine blue (TB) staining. We found numerous mast cells in the *Esr1*-deficient ovary in the interstitial area, whereas only singular mast cells were observed in WT ovaries (**Figure 7A**). This observation was reflected in the calculated number of mast cells (per mm^2^) in the ovaries. The mean calculated number of mast cells in 38.6 ± 11.5 mast cells per mm^2^ in *Esr1*-deficient ovary was significantly higher than those observed in WT ovaries (2.8 ± 4.3 mast cells per mm^2^) (**Figure 7B**). In addition, TB staining in *Esr1*-deficient ovaries showed yellow deposits that were located at the same sides of iron deposits found in PPB staining (**Figure 7C**). Interestingly, we observed some degranulated mast cells in the *Esr1*-deficient ovaries, indicated by purple-stained granules that were released in the surrounding ovarian tissue (**Figure 7D**). In line, RT-qPCR revealed significantly higher levels of mast cell protease 6 and 2 (*Mcpt6, Mcpt2)* in *Esr1*-depleted ovaries (**Figure 7E**).

Overall, the number of mast cells is increased in *Esr1*-deficient ovaries compared with WT animals, suggesting that ovarian mast cells play an active role in the hemorrhagic cystic *Esr1*-knockout phenotype and might be involved in the formation of MNGCs.

## 4 Discussion

Estradiol (E2) and estrogen receptor alpha (ERα, *Esr1*) are essential for the maintenance of normal ovarian function and development in mammals. Consequently, *Esr1-*deficient animals are infertile (1, 14, 17, 48). The role of ERα in aging, particularly in ovarian aging is poorly understood. A recent study gave evidence that hormonal imbalance leads to increased iron deposition and altered iron homeostasis (20).

Overall, our data show that *Esr1*-deficient ovaries exhibit signs that are comparable to those seen in ovarian aging. Most interesting is the massive iron overload in the *Esr1*-deficient ovaries, whereas only very small amounts are found in WT ovaries. The ovarian iron overload was confirmed by different techniques. We first demonstrated it by LA-ICP-MS imaging technique, which has already been established in hepatic metal bio-imaging for diagnostics (53, 71). LA-ICP-MS imaging revealed that *Esr1*-deficient ovaries had approximately 4-fold higher quanties of iron compared with WTs. Based on the light microscopy images of the native preparations and the LA-ICP-MS images that provided precise iron distribution within the tissue, we conclude that large iron deposits are localized in the ovarian stroma of the animals, which is in contrast to the normal ovary where it is present within the atrectic follicles or regressing *corpora lutea*. Due to anovulation, infertile *Esr1*-deficient mice develop large cysts in the ovaries, which are mainly hemorrhagic (14, 17, 48), suggesting a link or a cause for elevated iron. Since iron is required for a variety of physiological prcoesses, but toxic in free form, precise balance of iron metabolism is mandatory (28). However, LA-ICP-MS analysis is limited to the determination of the elemental content of biometals and not suitable to assess the electron configation of measured iron. Therefore, it was necessary to investigate the form of the severe iron excess in *Esr1*-deficient ovaries and how this affects the iron metabolism of the animals. Our data demonstrate that ovarian iron deposition in *Esr1*-deficient animals was a local and not a systemic condition, due to the fact that liver and spleen (which act as important organs for iron storage and recycling) (35) were similar in control and *Esr1*-deficient animals. For a more detailed analysis of the systemic effects of iron, it would be useful to perform a comprehensive serum analysis. This analysis should include measuring typical iron-related parameters such as unsaturated iron binding capacity, transferrin saturation, and hemoglobin.

In a previous study we demonstrated that *Esr1* deletion dramatically increases expression of lipocalin 2 (LCN2), which acts as an alternative iron transporter (8). In line, we here found that *Esr1*-deficient ovaries had significantly increased transferrin mRNA and protein expression whem compared to WT ovaries, which indicates enhanced iron transport, possibly due to the hemorrhaghic phenotype of the animals. The iron storage protein ferritin that exists either as heavy (Fth1) or as light chain (Ftl1), was strongly expressed by the murine ovaries, but only *Fth1* mRNA was significantly enhanced in *Esr1*-deficient ovaries. In addition, we found significantly higher expression of *Slc11a2*, a metal importer that is resposible for uptake of Fe^2+^ (72), in *Esr1*-depleted ovaries. Comparable, *Slc11a2* expression is elevated in ovarian endometriosis, a disease of the female reproductive tract associated with iron and related ovarian carcinogenesis (73). This suggests that the hemorrhagic iron-overload phenotype of *Esr1*-deficient ovaries has certain parallels with these diseases that are influenced by hormones. That only *Fth1* is strongly elevated in *Esr1*-deficient ovaries, might be explained by the functional difference between *Fth1* and *Ftl1*. It is known that only Fth1 has the ability to oxidize Fe^2+^ to Fe^3+^ (74). Considering that Slc11a2 mediates the uptake of iron as Fe^2+^, thereby leading to increase iron levels, enhanced *Fth*1 expression could be essential in protecting against iron-induced oxidative stress.

Iron-regulatory proteins such as IRP2 regulate iron metabolism post-transcriptionally. When iron is present, IRP2 loses its high affinity for RNA, leading to an increase ferritin to prevent cellular damage (74). In contrast, we observed increased *Ireb2* expression in *Esr1*-deficient ovaries compared to WT controls. An abberrant expression of IRPs, especially upregulation of IRP2, has been observed in aging rat ovaries and endometrial cysts (64, 75). These and our results underline that certain conditions, such as excess iron, upset the regulation of iron metabolism, which presumably leads to cellular damage in the long term.

Using PPB staining, we detected a tremendous accumulation of hemosiderin in *Esr1*-deficient ovaries, closely resembling the characteristics of aged ovaries (21, 23) In naturally aged murine ovaries, these deposits are stored in macrophages (so called hemosiderin-laden macrophages) as a consequence of red blood cell phagocytosis (21). It is likely that these cells are also present in *Esr1*-deficient ovary, as the hemorrhagic phenotype of these animals is clearly a source of these cells. However, it is still not clear what function and effect can be attributed to this deposition of hemosiderin in the tissue. Since hemosiderin is composed in part of degraded ferritin, it is postulated that hemosiderin acts as a protective factor in the cell by reducing the ability of iron to promote oxidative stress (33). On the other hand, it is assumed that decreased stability of hemosiderin deposits may lead to iron-induced lipid peroxidation in disease and aging (76). In addition, intramyocardial hemorrhages was shown to recruit iron-laden marcrophages in an animal model of myocardial infarction, which provokes foam cell formation and lipid peroxidation (66). Therefore, we concluded that the hemosiderin deposition in the ovaries is most likely due to the hemorrhagic phenotype of the *Esr1*-deficient animals and could probably be cell-damaging.

Iron overload with increased lipid peroxidation can lead to tissue damage resulting in ferroptosis, a form of oxidative damage-related cell death (77). However, there are a variety of mechanisms that can be activated to reduce severe cellular damage caused by *e.g.* excess iron. Interestingly, Fths function is not only limited to iron storage. It further has essential roles in the detoxification capacity of macrophages (78). Ferroptosis (iron-induced cell death) is characterized by ferritinophagy, the degradation of ferritins. However, expression of genes associated with ferroptosis were not found different in *Esr1*-deficient and WT ovaries. Another defense mechanism of ferroptosis is the expression of glutathione peroxidase 4 (GPX4) (79). The strong expression of *Gpx4* mRNA and protein in WT and *Esr1*-depleted ovaries suggests that antioxidant activity is not altered in ovaries lacking *Esr1*. In addition, we found that *Esr1*-deficient animals show significantly enhanced *Hmox1* mRNA expression. In general, activation cytoprotective enzymes such as heme oxygenase-1 (HMOX1, *Hmox1*) is a mechanism to handle iron overload conditions (35, 80). In aging female mice, HMOX1 expression was found around atrectic follicles in macrophages, indicating degeneration of engulfed heme (23). Overall, we can conclude from our data that defense mechanisms against oxidative stress are activated in the *Esr1*-deficient animals, while we observed no alterations in iron-driven cell death associated with the lack of *Esr1*. Since there are a large number of parameters associated with iron-induced cell damage, it is advisable to comprehensively investigate these in future studies. It would be suitable to examine the amount of 4-hydroxynonenal as a marker of lipid peroxidation and analyze the Nrf2-Keap1 axis, which is one of the most important signaling pathways in the control of oxidative and electrophilic stress. Additionally, it is important to not only focus on protein expression, but also on its localization in the tissue.

Since macrophages are recruited in hemorrhage conditions and are key players in iron metabolism, we investigated whether these are aberrant in the *Esr1*-deficient ovaries. Indeed, we found signifcantly higher expression of *Cd68* and *Adgre* representin important pan-macrophage markers. In line, immunofluoresence detection of F4/80 verified these findings. Interestingly, we found no co-localization of F4/80 and the iron regulator LCN2. LCN2 that plays a pleiotropic role in relation to macrophages and inflammation, depends on various factors (81, 82). Our results show significantly higher expression of M2-like macrophage markers (*Arg1*, *Cd163*, *Mrc*) in *Esr1*-deficient ovaries when compared to WT animals, whereas, contrary to expectations, there was no significant difference in M1-like markers (*Il1r*, *Nos2*). These results suggest that despite the intense iron accumulation, the ovary of *Esr1*-knockout animals express anti-inflammatory markers, which are indicative for remodeling and tissue repair, similar to characteristics foun during ovarian aging (38). Further studies should investigate systemic levels of inflammatory markers and clearly identify whether these ovarian immune cells are resident or infiltrating.

In the last decade, a unique type of ovarian macrophage found exclusively in reproductively old animals was discovered (21–23, 34). Based on the typical appearance of this cell type, these cells are referred as MNGCs. These cells are likely the result of a disruption in normal physiology that is caused by altered endocrinology as one possible cause (34). Our data provides the first evidence that endocrine dysfunction indeed causes formation of MNGCs, as these cells are present in the aged ovaries of *Esr1*-deficient animals but not in WT littermates. Considering that their appearance correlates with a strong increase in classical M2-like macrophage markers, they may play a role in remodeling and tissue repair. In addition, we found that iron storage seems to play a role in these cells, as we confirmed iron deposition in the MNGCs by PPB staining. These foam cell clusters in *Esr1*-deficient ovaries exhibit strong autofluroresence, likely due to accumulation of lipofuscin, an ‘aging-pigment observed in ovarian aging and disease (21–23, 83). Lipofuscin and iron storage are associated with oxidative DNA damage and ovarian disease (21). Although our limited results suggest that adult *Esr1*-deficient animals have not higher tendency to induce ferroptosis than WT animals, it is possible that this changes after futher aging. This is supported by preliminary data of a small number of older *Esr1*-deficient animals that we have analyzed, showing strong accumulation of lipofuscin and massive iron deposits in the aged organs (cf. **Supplementary Figure 3**). Interestingly, preliminary (unpublished) results using Oil Red staining show that the MNGCs in *Esr1*-deficient ovaries and aged WT ovaries contain numerous lipid droplets. Further analysis of these specific MNGCs, which are formed in both the aged WT animals and after *Esr1* depletion, will shed light on the potential link between aging processes and *Esr1* signaling.

Studies outside the ovary have shown that iron-induced macrophage influx and foam cell formation in a hemorrhage lesion are associated with increased mast cell activity (66). In fact, our results confirm a similar finding in the hemorrhagic ovary as there is a significantly higher number of mast cells in *Esr1*-deficient than in WT ovaries. This is consistent with the results of another *Esr1* depletion model, in which the PCOS phenotype is associated with enhanced mast cells (13). Interestingly, release of granules from mast cells is mediated by hormones such as luteinizing hormone (84). As suggested previously by others (13), high quantities of serum luteinizing hormone in the *Esr1*-deficient ovary (48) may lead to mast cell activity.

## 5 Conclusion

In the study presented, we have demonstrated that disruption of *Esr1* in the mouse ovary leads to changes in iron metabolism. Specifically, we report for the first time an iron overload phenotype in the *Esr1*-deficient ovary, accompanied by abnormal iron metabolism. In addition, these animals showed increased numbers of ovarian macrophages and mast cells. Notably, we observed an influx of M2-like macrophages and formation of foam macrophages, which are considered hallmarks of ovarian aging. Therefore, our results suggest that there may be similar underlying mechanisms involved in ovarian aging. However, further studies, including age-dependent observation of *Esr1*-deficient animals are required to understand the role and potential consequences of these MNGCs in the ovary. In this context, it is also important to determine whether tissue architecture remodeling is abnormal in *Esr1*-deficient ovaries, as is seen in aging (22, 85). A better understanding of the aging processes in the ovaries could therefore lead to the development of therapies that curb oocyte damage and age-related infertility.

## Data availability statement

The raw data supporting the conclusions of this article will be made available by the authors, without undue reservation.

## Ethics statement

The animal study was approved by internal Review Board of the RWTH University Hospital Aachen. The study was conducted in accordance with the local legislation and institutional requirements.

## Author contributions

SKS: Conceptualization, Formal analysis, Investigation, Methodology, Project administration, Validation, Visualization, Writing – original draft. MK: Formal analysis, Investigation, Methodology, Validation, Visualization, Writing – review & editing. PK: Formal analysis, Methodology, Visualization, Writing – review & editing. JCK: Formal analysis, Methodology, Writing – review & editing. RW: Conceptualization, Data curation, Funding acquisition, Investigation, Project administration, Resources, Supervision, Visualization, Writing – review & editing.

## Glossary

**Table:**

Aco1	Aconitase 1
Adgre	Adhesion G protein-coupled receptor
Arg1	Arginase 1
Cd	Cluster of differentiation
E2	17β-Estradiol
ER, Esr	Estrogen receptor
Fpn	Ferroportin
Fth1	Ferritin heavy chain 1
Ftl1	Ferritin light chain 1
GPER1	G-protein coupled estrogen receptor 1
Hamp1	Hepcidin antimicrobial peptide 1
HE	Hematoxylin-eosin
Il1r1	Interleukin 1 receptor type 1
IRP	Iron regulatory protein
LA-ICP-MS	laser ablation inductively coupled plasma mass spectrometry
LCN2	Lipocalin 2
Mcpt	Mast cell protease
MNGC	Macrophage-derived multinucleated giant cell
Mrc	Mannose receptor
Nos2	Nitric oxide synthase 2
PAS	Periodic acid-Schiff
PBS	Phosphate-buffered saline
PBS-T	PBS supplemented with 0.1% Tween^®^ 20
PCOS	Polycystic ovary syndrome
PPB	Perls Prussian Blue
RT	Room temperature
RT-qPCR	reverse transcription and quantitative real-time polymerase chain reaction
Slc11a2	Solute carrier family 11 member 2
TB	Toluidine Blue
Tf	Transferrin

## References

[1] Hamilton KJ Hewitt SC Arao Y Korach KS. Estrogen hormone biology. Curr Top Dev Biol (2017) 125:109–46. doi: 10.1016/bs.ctdb.2016.12.005 PMC6206851 28527569

[2] Mahboobifard F Pourgholami MH Jorjani M Dargahi L Amiri M Sadeghi S. Estrogen as a key regulator of energy homeostasis and metabolic health. BioMed Pharmacother (2022) 156:113808. doi: 10.1016/j.biopha.2022.113808 36252357

[3] Hewitt SC Korach KS. Estrogen receptors: new directions in the new millennium. Endocrine Rev (2018) 39:664–75. doi: 10.1210/er.2018-00087 PMC6173474 29901737

[4] Schroder SK Tag CG Kessel JC Antonson P Weiskirchen R. Immunohistochemical detection of estrogen receptor-beta (ERβ) with PPZ0506 antibody in murine tissue: from pitfalls to optimization. Biomedicines (2022) 10:3100. doi: 10.3390/biomedicines10123100 36551855 PMC9775465

[5] Kuiper GG Enmark E Pelto-Huikko M Nilsson S Gustafsson JA. Cloning of a novel receptor expressed in rat prostate and ovary. Proc Natl Acad Sci U S A (1996) 93:5925–30. doi: 10.1073/pnas.93.12.5925 PMC39164 8650195

[6] Hiroi H Inoue S Watanabe T Goto W Orimo A Momoeda M. Differential immunolocalization of estrogen receptor alpha and beta in rat ovary and uterus. J Mol Endocrinology (1999) 22:37–44. doi: 10.1677/jme.0.0220037 9924178

[7] Sar M Welsch F. Differential expression of estrogen receptor-β and estrogen receptor-α in the rat ovary. Endocrinology (1999) 140:963–71. doi: 10.1210/endo.140.2.6533 9927330

[8] Kessel JC Weiskirchen R Schroder SK. Expression analysis of lipocalin 2 (LCN2) in reproductive and non-reproductive tissues of esr1-deficient mice. Int J Mol Sci (2023) 24:9280. doi: 10.3390/ijms24119280 37298232 PMC10252910

[9] Nalvarte I Antonson P. Estrogen receptor knockout mice and their effects on fertility. Receptors (2023) 2:116–26. doi: 10.3390/receptors2010007

[10] Couse JF Korach KS. Estrogen receptor null mice: what have we learned and where will they lead us? Endocrine Rev (1999) 20:358–417. doi: 10.1210/edrv.20.3.0370 10368776

[11] Hamilton KJ Arao Y Korach KS. Estrogen hormone physiology: reproductive findings from estrogen receptor mutant mice. Reprod Biol (2014) 14:3–8. doi: 10.1016/j.repbio.2013.12.002 24607249 PMC4777324

[12] Hewitt SC Couse JF Korach KS. Estrogen receptor transcription and transactivation: Estrogen receptor knockout mice: what their phenotypes reveal about mechanisms of estrogen action. Breast Cancer Res (2000) 2:345–52. doi: 10.1186/bcr79 PMC138656 11250727

[13] Dupont S Krust A Gansmuller A Dierich A Chambon P Mark M. Effect of single and compound knockouts of estrogen receptors alpha (ERalpha) and beta (ERbeta) on mouse reproductive phenotypes. Development (2000) 127:4277–91. doi: 10.1242/dev.127.19.4277 10976058

[14] Lubahn DB Moyer JS Golding TS Couse JF Korach KS Smithies O. Alteration of reproductive function but not prenatal sexual development after insertional disruption of the mouse estrogen receptor gene. Proc Natl Acad Sci United States America (1993) 90:11162–6. doi: 10.1073/pnas.90.23.11162 PMC47942 8248223

[15] Antonson P Apolinario LM Shamekh MM Humire P Poutanen M Ohlsson C. Generation of an all-exon Esr2 deleted mouse line: Effects on fertility. Biochem Biophys Res Commun (2020) 529:231–7. doi: 10.1016/j.bbrc.2020.06.063 32703416

[16] Prossnitz ER Hathaway HJ. What have we learned about GPER function in physiology and disease from knockout mice? J Steroid Biochem Mol Biol (2015) 153:114–26. doi: 10.1016/j.jsbmb.2015.06.014 PMC4568147 26189910

[17] Schomberg DW Couse JF Mukherjee A Lubahn DB Sar M Mayo KE. Targeted disruption of the estrogen receptor-alpha gene in female mice: characterization of ovarian responses and phenotype in the adult. Endocrinology (1999) 140:2733–44. doi: 10.1210/endo.140.6.6823 10342864

[18] Britt KL Drummond AE Dyson M Wreford NG Jones ME Simpson ER. The ovarian phenotype of the aromatase knockout (ArKO) mouse. J Steroid Biochem Mol Biol (2001) 79:181–5. doi: 10.1016/S0960-0760(01)00158-3 11850223

[19] Britt KL Drummond AE Cox VA Dyson M Wreford NG Jones ME. An age-related ovarian phenotype in mice with targeted disruption of the Cyp 19 (aromatase) gene. Endocrinology (2000) 141:2614–23. doi: 10.1210/endo.141.7.7578 10875266

[20] Toothaker JM Roosa K Voss A Getman SM Pepling ME. Oocyte survival and development during follicle formation and folliculogenesis in mice lacking aromatase. Endocrine Res (2022) 47:45–55. doi: 10.1080/07435800.2021.2011907 34866531

[21] Urzua U Chacon C Espinoza R Martinez S Hernandez N. Parity-dependent hemosiderin and lipofuscin accumulation in the reproductively aged mouse ovary. Anal Cell Pathol (Amst) (2018) 2018:1289103. doi: 10.1155/2018/1289103 29736365 PMC5874974

[22] Briley SM Jasti S McCracken JM Hornick JE Fegley B Pritchard MT. Reproductive age-associated fibrosis in the stroma of the mammalian ovary. Reproduction (2016) 152:245–60. doi: 10.1530/REP-16-0129 PMC4979755 27491879

[23] Asano Y. Age-related accumulation of non-heme ferric and ferrous iron in mouse ovarian stroma visualized by sensitive non-heme iron histochemistry. J Histochem Cytochem (2012) 60:229–42. doi: 10.1369/0022155411431734 PMC3351130 22108647

[24] Mathew M Sivaprakasam S Phy JL Bhutia YD Ganapathy V. Polycystic ovary syndrome and iron overload: biochemical link and underlying mechanisms with potential novel therapeutic avenues. Biosci Rep (2023) 43:BSR20212234. doi: 10.1042/BSR20212234 36408981 PMC9867939

[25] Escobar-Morreale HF. Iron metabolism and the polycystic ovary syndrome. Trends Endocrinol Metab (2012) 23:509–15. doi: 10.1016/j.tem.2012.04.003 22579050

[26] Sanchez AM Papaleo E Corti L Santambrogio P Levi S Vigano P. Iron availability is increased in individual human ovarian follicles in close proximity to an endometrioma compared with distal ones. Hum Reprod (2014) 29:577–83. doi: 10.1093/humrep/det466 24430779

[27] Miller EM. The reproductive ecology of iron in women. Am J Phys Anthropol (2016) 159:S172–95. doi: 10.1002/ajpa.22907 26808104

[28] Galaris D Pantopoulos K. Oxidative stress and iron homeostasis: mechanistic and health aspects. Crit Rev Clin Lab Sci (2008) 45:1–23. doi: 10.1080/10408360701713104 18293179

[29] Xia L Shen Y Liu S Du J. Iron overload triggering ECM-mediated Hippo/YAP pathway in follicle development: a hypothetical model endowed with therapeutic implications. Front Endocrinol (Lausanne) (2023) 14:1174817. doi: 10.3389/fendo.2023.1174817 37223010 PMC10200985

[30] Anderson GJ McLaren GD. Iron Physiology and Pathophysiology in Humans Humana Press, Springer New York, Dordrecht, Heidelberg, London (2012). doi: 10.1007/978-1-60327-485-2

[31] Cook SF. The structure and composition of hemosiderin. J Biol Chem (1929) 82:595–609. doi: 10.1016/S0021-9258(18)77144-5

[32] Saito H. Metabolism of iron stores. Nagoya J Med science (2014) 76:235–54. PMC4345694 25741033

[33] Iancu TC. Ferritin and hemosiderin in pathological tissues. Electron microscopy Rev (1992) 5:209–29. doi: 10.1016/0892-0354(92)90011-E 1581551

[34] Foley KG Pritchard MT Duncan FE. Macrophage-derived multinucleated giant cells: hallmarks of the aging ovary. Reproduction (2021) 161:V5–9. doi: 10.1530/REP-20-0489 PMC7856073 33258461

[35] Sukhbaatar N Weichhart T. Iron regulation: macrophages in control. Pharm (Basel) (2018) 11:137. doi: 10.3390/ph11040137 PMC6316009 30558109

[36] Zhang D Yu Y Duan T Zhou Q. The role of macrophages in reproductive-related diseases. Heliyon (2022) 8:e11686. doi: 10.1016/j.heliyon.2022.e11686 36468108 PMC9713353

[37] Zhang Z Huang L Brayboy L. Macrophages: an indispensable piece of ovarian health. Biol Reprod (2021) 104:527–38. doi: 10.1093/biolre/ioaa219 PMC7962765 33274732

[38] Zhang Z Schlamp F Huang L Clark H Brayboy L. Inflammaging is associated with shifted macrophage ontogeny and polarization in the aging mouse ovary. Reproduction (2020) 159:325–37. doi: 10.1530/REP-19-0330 PMC7066623 31940276

[39] Berger T Togawa A Duncan GS Elia AJ You-Ten A Wakeham A. Lipocalin 2-deficient mice exhibit increased sensitivity to Escherichia coli infection but not to ischemia-reperfusion injury. Proc Natl Acad Sci United States America (2006) 103:1834–9. doi: 10.1073/pnas.0510847103 PMC1413671 16446425

[40] Mosialou I Shikhel S Luo N Petropoulou PI Panitsas K Bisikirska B. Lipocalin-2 counteracts metabolic dysregulation in obesity and diabetes. J Exp Med (2020) 217:e20191261. doi: 10.1084/jem.20191261 32639539 PMC7537391

[41] Kjeldsen L Johnsen AH Sengelov H Borregaard N. Isolation and primary structure of NGAL, a novel protein associated with human neutrophil gelatinase. J Biol Chem (1993) 268:10425–32. doi: 10.1016/S0021-9258(18)82217-7 7683678

[42] Goetz DH Holmes MA Borregaard N Bluhm ME Raymond KN Strong RK. The neutrophil lipocalin NGAL is a bacteriostatic agent that interferes with siderophore-mediated iron acquisition. Mol Cell (2002) 10:1033–43. doi: 10.1016/S1097-2765(02)00708-6 12453412

[43] Flo TH Smith KD Sato S Rodriguez DJ Holmes MA Strong RK. Lipocalin 2 mediates an innate immune response to bacterial infection by sequestrating iron. Nature (2004) 432:917–21. doi: 10.1038/nature03104 15531878

[44] Xiao X Yeoh BS Vijay-Kumar M. Lipocalin 2: an emerging player in iron homeostasis and inflammation. Annu Rev Nutr (2017) 37:103–30. doi: 10.1146/annurev-nutr-071816-064559 28628361

[45] Rockfield S Raffel J Mehta R Rehman N Nanjundan M. Iron overload and altered iron metabolism in ovarian cancer. Biol Chem (2017) 398:995–1007. doi: 10.1515/hsz-2016-0336 28095368 PMC5545069

[46] Li A Ni Z Zhang J Cai Z Kuang Y Yu C. Transferrin insufficiency and iron overload in follicular fluid contribute to oocyte dysmaturity in infertile women with advanced endometriosis. Front Endocrinol (Lausanne) (2020) 11:391. doi: 10.3389/fendo.2020.00391 32636803 PMC7317002

[47] Zhang J Liu Y Yao W Li Q Liu H Pan Z. Initiation of follicular atresia: gene networks during early atresia in pig ovaries. J Reproduction (2018) 156:23–33. doi: 10.1530/REP-18-0058 29743261

[48] Hewitt SC Kissling GE Fieselman KE Jayes FL Gerrish KE Korach KS. Biological and biochemical consequences of global deletion of exon 3 from the ER alpha gene. FASEB J (2010) 24:4660–7. doi: 10.1096/fj.10.163428 PMC2992373 20667977

[49] Meguro R Asano Y Odagiri S Li C Iwatsuki H Shoumura K. Nonheme-iron histochemistry for light and electron microscopy: a historical, theoretical and technical review. Arch Histol cytology (2007) 70:1–19. doi: 10.1679/aohc.70.1 17558140

[50] Sobolev SM. Result of histochemical studies on certain PAS-positive substances in macrophages. Biulleten' eksperimental'noi biologii i meditsiny (1959) 47:104–9. doi: 10.1007/BF00779693 13671051

[51] Grigorev IP Korzhevskii DE. Modern imaging technologies of mast cells for biology and medicine (Review). Sovremennye tekhnologii v meditsine (2021) 13:93–107. doi: 10.17691/stm2021.13.4.10 PMC8482833 34603768

[52] Whittington NC Wray S. Suppression of red blood cell autofluorescence for immunocytochemistry on fixed embryonic mouse tissue. Curr Protoc Neurosci (2017) 81:2.28.1–2.12. doi: 10.1002/cpns.35 PMC5657453 29058770

[53] Kim P Weiskirchen S Uerlings R Kueppers A Stellmacher F Viveiros A. Quantification of liver iron overload disease with laser ablation inductively coupled plasma mass spectrometry. BMC Med Imaging (2018) 18:51. doi: 10.1186/s12880-018-0291-3 30514216 PMC6278171

[54] Chakrabarti M Cockrell AL Park J McCormick SP Lindahl LS Lindahl PA. Speciation of iron in mouse liver during development, iron deficiency, IRP2 deletion and inflammatory hepatitis. Metallomics (2015) 7:93–101. doi: 10.1039/C4MT00215F 25325718 PMC4276432

[55] Becker JS. Imaging of metals in biological tissue by laser ablation inductively coupled plasma mass spectrometry (LA-ICP-MS): state of the art and future developments. J Mass Spectrom (2013) 48:255–68. doi: 10.1002/jms.3168. 23412982

[56] Weiskirchen R Uerlings R. Laser ablation inductively coupled plasma mass spectrometry in biomedicine and clinical diagnosis. Cell Mol Medicine: Open access (2015) 01:1. doi: 10.21767/2573-5365 PMC4395195 25704483

[57] Uerlings R Matusch A. Reconstruction of laser ablation inductively coupled plasma mass spectrometry (LA-ICP-MS) spatial distribution images in Microsoft Excel 2007. Int Journals Mass Spectrometry (2016) 395:27–35. doi: 10.1016/j.ijms.2015.11.010

[58] Weiskirchen R Weiskirchen S Kim P Winkler R. Software solutions for evaluation and visualization of laser ablation inductively coupled plasma mass spectrometry imaging (LA-ICP-MSI) data: a short overview. J Cheminform (2019) 11:16. doi: 10.1186/s13321-019-0338-7 30778692 PMC6690067

[59] Boaru SG Merle U Uerlings R Zimmermann A Flechtenmacher C Willheim C. Laser ablation inductively coupled plasma mass spectrometry imaging of metals in experimental and clinical Wilson's disease. J Cell Mol Med (2015) 19:806–14. doi: 10.1111/jcmm.12497 PMC4395195 25704483

[60] Kim P Zhang CC Thoroe-Boveleth S Buhl EM Weiskirchen S Stremmel W. Analyzing the therapeutic efficacy of bis-choline-tetrathiomolybdate in the atp7b(-/-) copper overload mouse model. Biomedicines (2021) 9:1861. doi: 10.3390/biomedicines9121861 34944677 PMC8698685

[61] Pathak P Kapil U. Role of trace elements zinc, copper and magnesium during pregnancy and its outcome. Indian J pediatrics (2004) 71:1003–5. doi: 10.1007/BF02828116 15572821

[62] Ceko MJ O'Leary S Harris HH Hummitzsch K Rodgers RJ. Trace elements in ovaries: measurement and physiology. Biol Reprod (2016) 94:86. doi: 10.1095/biolreprod.115.137240 26864198

[63] Kovtunovych G Eckhaus MA Ghosh MC Ollivierre-Wilson H Rouault TA. Dysfunction of the heme recycling system in heme oxygenase 1-deficient mice: effects on macrophage viability and tissue iron distribution. Blood (2010) 116:6054–62. doi: 10.1182/blood-2010-03-272138 PMC3031391 20844238

[64] Sze SCW Zhang L Zhang S Lin K Ng TB Ng ML. Aberrant transferrin and ferritin upregulation elicits iron accumulation and oxidative inflammaging causing ferroptosis and undermines estradiol biosynthesis in aging rat ovaries by upregulating NF-kappab-activated inducible nitric oxide synthase: first demonstration of an intricate mechanism. Int J Mol Sci (2022) 23:12689. doi: 10.3390/ijms232012689 36293552 PMC9604315

[65] Kasztura M Kiczak L Paslawska U Bania J Janiszewski A Tomaszek A. Hemosiderin accumulation in liver decreases iron availability in tachycardia-induced porcine congestive heart failure model. Int J Mol Sci (2022) 23:1026. doi: 10.3390/ijms23031026 35162949 PMC8834801

[66] Cokic I Chan SF Guan X Nair AR Yang HJ Liu T. Intramyocardial hemorrhage drives fatty degeneration of infarcted myocardium. Nat Commun (2022) 13:6394. doi: 10.1038/s41467-022-33776-x 36302906 PMC9613644

[67] Zhang Y Ramos BF Jakschik BA. Neutrophil recruitment by tumor necrosis factor from mast cells in immune complex peritonitis. Sci (New York NY) (1992) 258:1957–9. doi: 10.1126/science.1470922 1470922

[68] Batth BK Parshad RK. Mast cell dynamics in the house rat (Rattus rattus) ovary during estrus cycle, pregnancy and lactation. Eur J morphology (2000) 38:17–23. doi: 10.1076/0924-3860(200002)38:01;1-#;FT017 10550797

[69] Karaca T Yoruk M Uslu S. Distribution and quantitative patterns of mast cells in ovary and uterus of rat. Archivos medicina veterinaria (2007) 39:135–39. doi: 10.4067/S0301-732X2007000200006

[70] Atiakshin D Patsap O Kostin A Mikhalyova L Buchwalow I Tiemann M. Mast cell tryptase and carboxypeptidase A3 in the formation of ovarian endometrioid cysts. Int J Mol Sci (2023) 24:6498. doi: 10.3390/ijms24076498 37047472 PMC10095096

[71] Weiskirchen S Kim P Weiskirchen R. Determination of copper poisoning in Wilson's disease using laser ablation inductively coupled plasma mass spectrometry. Ann Transl Med (2019) 7:S72. doi: 10.21037/atm 31179309 PMC6531650

[72] Vogt AS Arsiwala T Mohsen M Vogel M Manolova V Bachmann MF. On iron metabolism and its regulation. Int J Mol Sci (2021) 22:4591. doi: 10.3390/ijms22094591 33925597 PMC8123811

[73] Akashi K Nagashima Y Tabata T Oda H. Immunochemical analysis of iron transporters and M2 macrophages in ovarian endometrioma and clear cell adenocarcinoma. Mol Clin Oncol (2021) 15:159. doi: 10.3892/mco 34194738 PMC8237161

[74] Wallander ML Leibold EA Eisenstein RS. Molecular control of vertebrate iron homeostasis by iron regulatory proteins. Biochim Biophys Acta (2006) 1763:668–89. doi: 10.1016/j.bbamcr.2006.05.004 PMC2291536 16872694

[75] Takenaka M Suzuki N Mori M Hirayama T Nagasawa H Morishige KI. Iron regulatory protein 2 in ovarian endometrial cysts. Biochem Biophys Res Commun (2017) 487:789–94. doi: 10.1016/j.bbrc.2017.04.115 28450115

[76] Tian Y Tian Y Yuan Z Zeng Y Wang S Fan X. Iron metabolism in aging and age-related diseases. Int J Mol Sci (2022) 23:3612. doi: 10.3390/ijms23073612 35408967 PMC8998315

[77] Chen X Comish PB Tang D Kang R. Characteristics and biomarkers of ferroptosis. Front Cell Dev Biol (2021) 9:637162. doi: 10.3389/fcell.2021.637162 33553189 PMC7859349

[78] Mesquita G Silva T Gomes AC Oliveira PF Alves MG Fernandes R. H-Ferritin is essential for macrophages' capacity to store or detoxify exogenously added iron. Sci Rep (2020) 10:3061. doi: 10.1038/s41598-020-59898-0 32080266 PMC7033252

[79] Zhang LL Tang RJ Yang YJ. The underlying pathological mechanism of ferroptosis in the development of cardiovascular disease. Front Cardiovasc Med (2022) 9:964034. doi: 10.3389/fcvm.2022.964034 36003910 PMC9393259

[80] Recalcati S Cairo G. Macrophages and iron: a special relationship. Biomedicines (2021) 9:1585. doi: 10.3390/biomedicines9111585 34829813 PMC8615895

[81] An HS Yoo JW Jeong JH Heo M Hwang SH Jang HM. Lipocalin-2 promotes acute lung inflammation and oxidative stress by enhancing macrophage iron accumulation. Int J Biol Sci (2023) 19:1163–77. doi: 10.7150/ijbs.79915 PMC10008694 36923935

[82] Jung M Brune B Hotter G Sola A. Macrophage-derived Lipocalin-2 contributes to ischemic resistance mechanisms by protecting from renal injury. Sci Rep (2016) 6:21950. doi: 10.1038/srep21950 26911537 PMC4766505

[83] Watson JM Marion SL Rice PF Utzinger U Brewer MA Hoyer PB. Two-photon excited fluorescence imaging of endogenous contrast in a mouse model of ovarian cancer. Lasers Surg Med (2013) 45:155–66. doi: 10.1002/lsm.22115 PMC4566968 23362124

[84] Norman RJ Brannstrom M. White cells and the ovaryincidental invaders or essential effectors? J Endocrinol (1994) 140:333–6. doi: 10.1677/joe.0.1400333 8182359

[85] Amargant F Manuel SL Tu Q Parkes WS Rivas F Zhou LT. Ovarian stiffness increases with age in the mammalian ovary and depends on collagen and hyaluronan matrices. Aging Cell (2020) 19:e13259. doi: 10.1111/acel.13259 33079460 PMC7681059

