# Supplementary material for: Ovaries of estrogen receptor 1-deficient mice show iron overload and signs of aging
Source: Front Endocrinol (Lausanne). 2024 Feb 23;15:1325386. doi: 10.3389/fendo.2024.1325386 (PMC10920212; doi:10.3389/fendo.2024.1325386)
Supplement: Supplementary file 1 [file DataSheet_1.pdf]

Supplementary Material

Ovaries of Estrogen receptor 1-Deficient Mice Show Iron Overload and Accelerated Aging

Sarah K. Schröder[1*], Marinela Krizanac[1], Philipp Kim[1], Jan C. Kessel[1], Ralf Weiskirchen[1*]

[1]Institute of Molecular Pathobiochemistry, Experimental Gene Therapy and Clinical Chemistry (IFMPEGKC), RWTH University Hospital Aachen, D-52074 Aachen, Germany

*** Correspondence:**
Corresponding Authors
saschroeder@ukaachen.de; rweiskirchen@ukaachen.de

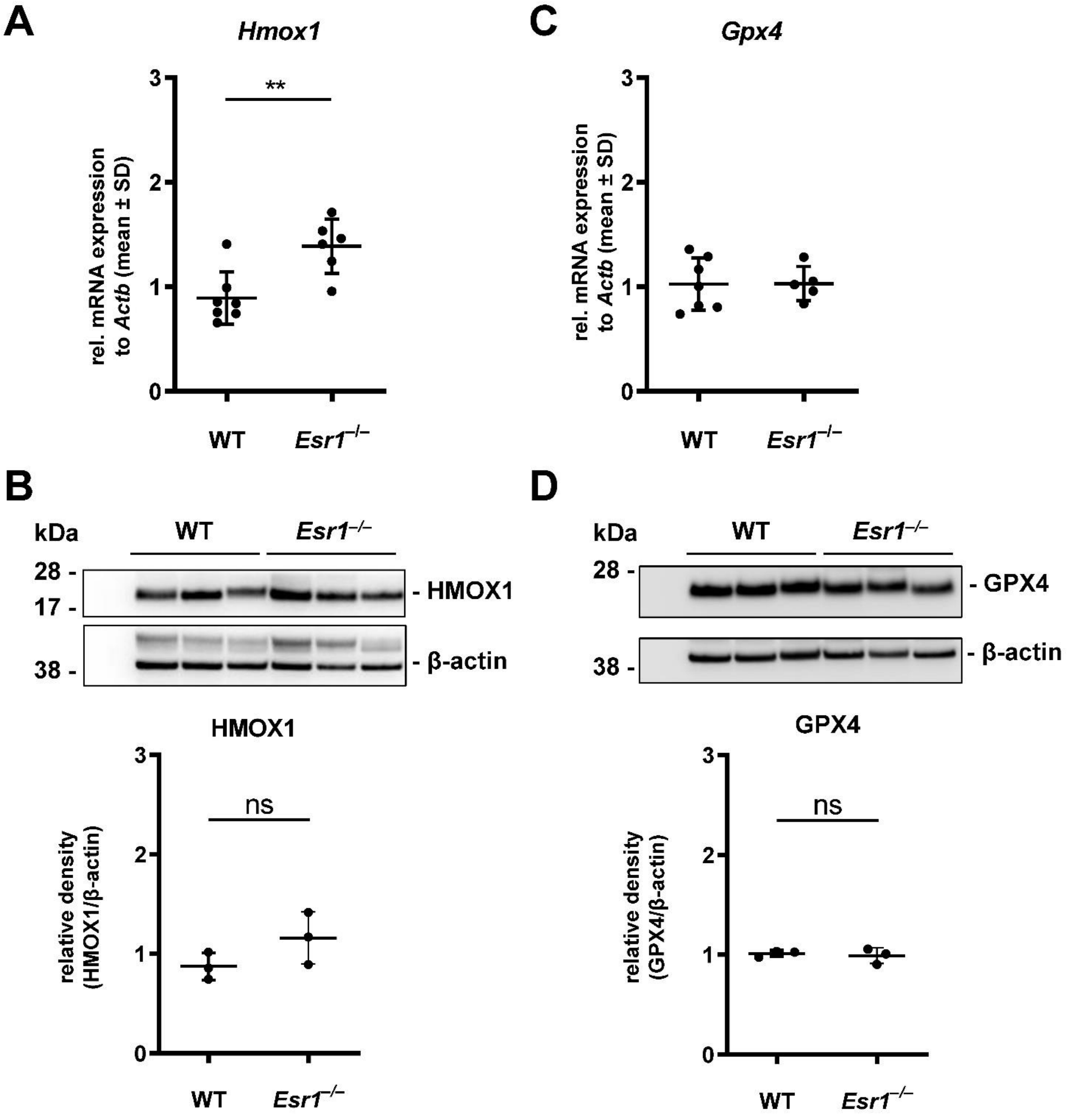

Suppl. Figure 1: Oxidative defense in ovarian tissue. Wild type (WT) and *Esr1*-deficient ovarian tissues were used for RNA (WT, n=7; *Esr1*$^{-/-}$, n=5) and Western blot analysis (WT, n=3; *Esr1*$^{-/-}$, n=3). (**A**) Relative *Hmox1* mRNA was measured by RT-qPCR and (**B**) HMOX1 protein expression was determined by Western blot analysis, quantified densitometrically and plotted relative to β-actin expression. (**C**) Relative *Gpx4* mRNA was measured by RT-qPCR and (**B**) GPX4 protein expression was determined by Western blot analysis, quantified densitometrically and plotted relative to β-actin expression. All data (**A-D**) are displayed as mean ± SD. For statistical analysis a Student's *t*-test was done. Significant differences between groups are marked with asterisks: ** p<0.01, ns = not significant.

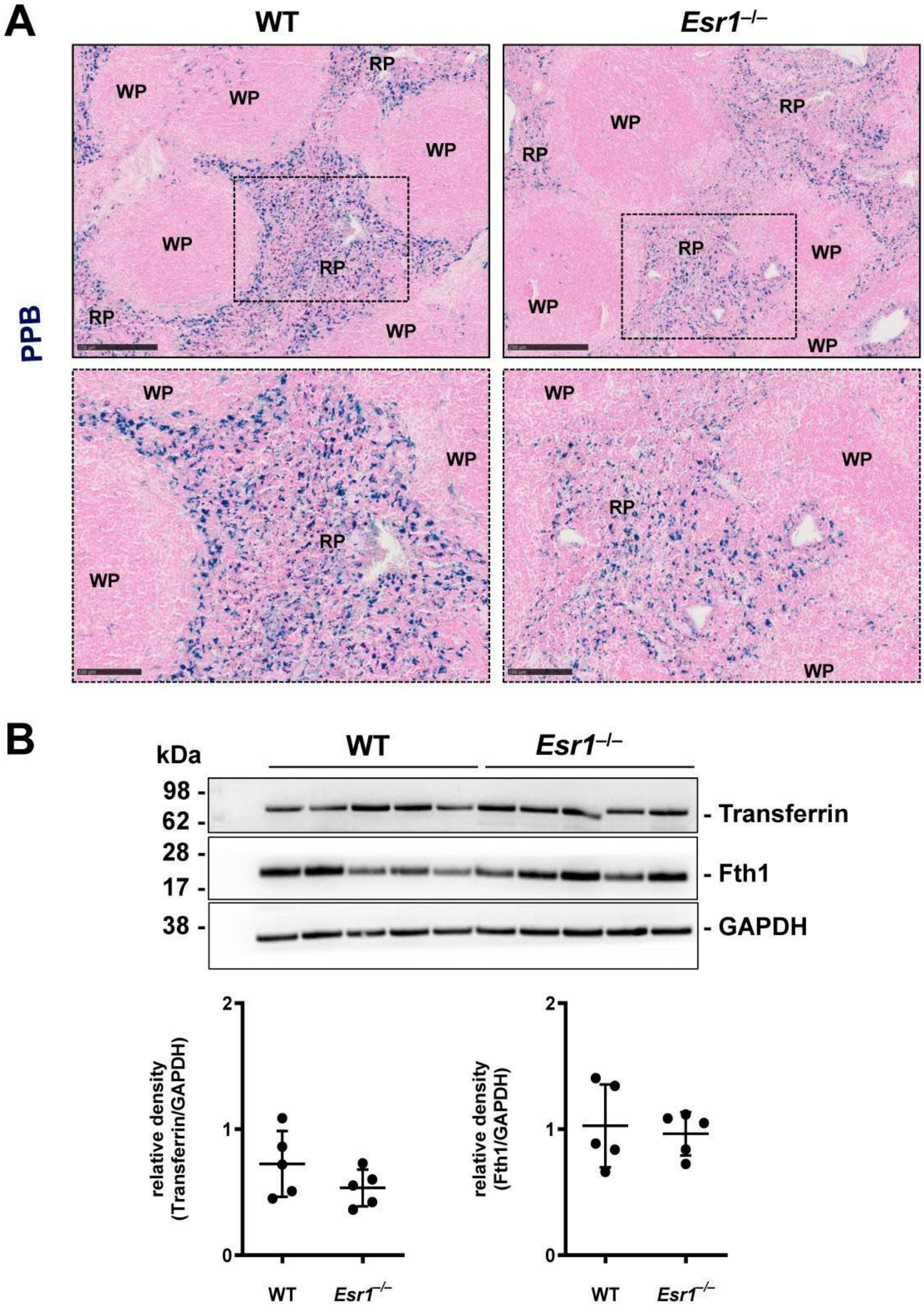

Suppl. Figure 2: Cellular iron metabolism in spleen tissue. Wild type (WT) and *Esr1*-deficient spleen tissues were used for staining or Western blot analysis. (**A**) Formalin-fixated paraffin-embedded liver tissue sections (WT, n=5; *Esr1*$^{-/-}$, n=5) were stained for iron using Perls Prussian Blue (PPB). The scale bars correspond to 250 µm or 100 µm in the partial section (dashed boarder), respectively. (**B**) Protein expression of transferrin and Fth1 was investigated by Western blot analysis (WT, n=5; *Esr1*$^{-/-}$, n=5). Expression levels were quantified densitometrically and plotted relative to GADH expression. Data is displayed as mean ± SD. For statistical analysis a Student's *t*-test was done. No significant differences were found between the groups. Abbreviations used are: RP, red pulp; WP, white pulp.

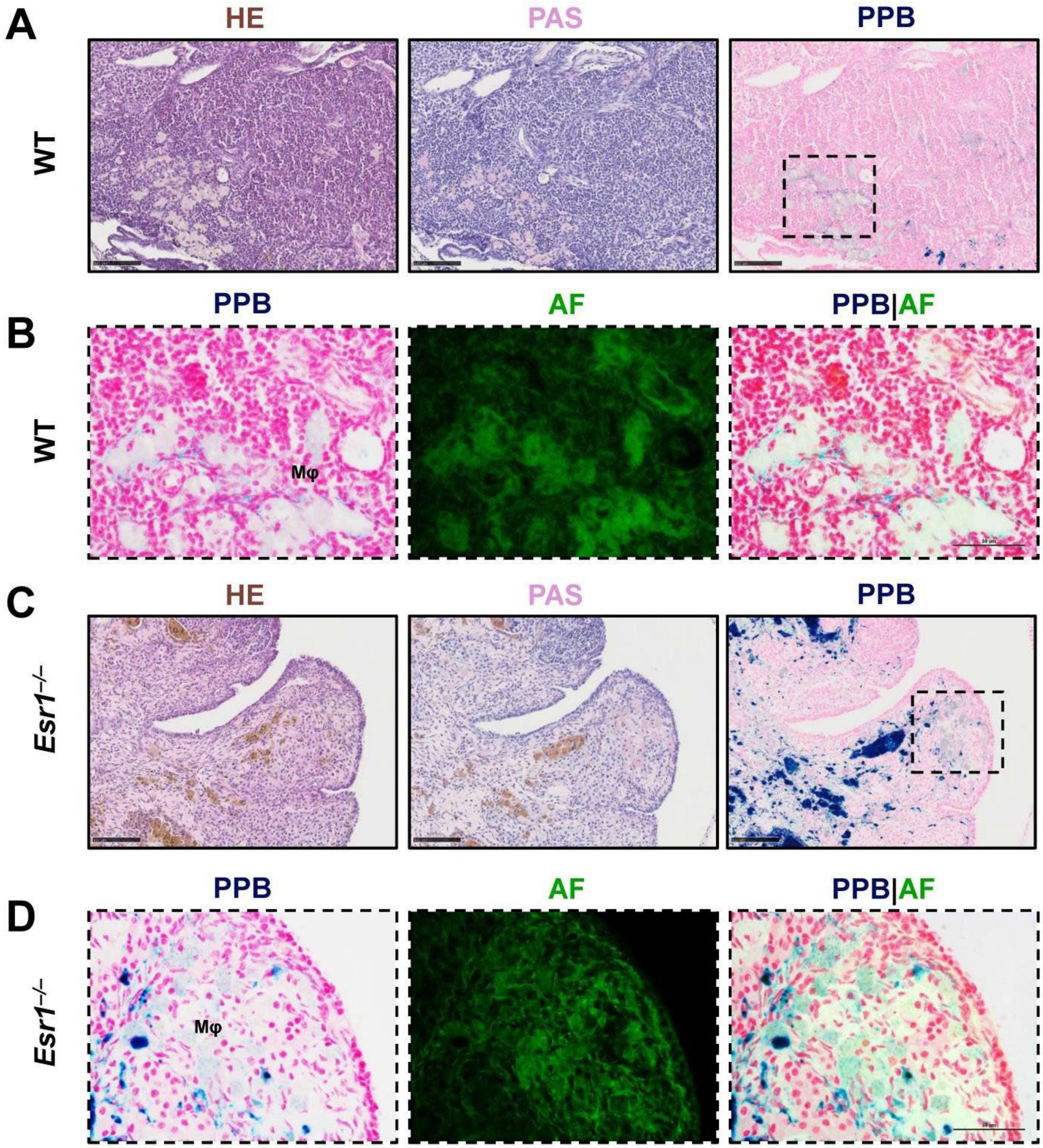

Suppl. Figure 3: Macrophage-derived multinucleated giant cells (MNGCs) in aged wild type (WT) and *Esr1*-deficient ovaries. Ovarian tissues were dissected from 18-month-old animals (WT, n=3; *Esr1*$^{–/–}$, n=2) and stained with Hematoxylin-Eosin (HE), Periodic Acid Schiff (PAS), and Perls Prussian Blue (PPB). Clusters of MNGCs were observed in WT **(A,B)** and *Esr1*-deficient **(C,D)** ovaries. In addition, MNGCs in PPB-stained tissue sections exhibited strong autofluorescence, indicating accumulation of lipofuscin in both genotypes **(B,D)**. Scale bars correspond to 100 µm **(A,C)** or 50 µm in the partial section (dashed boarder) in (**B,D**), respectively. Mφ, macrophage.

Suppl. Table 1: List of Primers

Mouse Gene	Accession No.	Primer (5'-3')
Aco1	NM_007386.2	For: ggacatcgtgctcaccattac<br>Rev: tgcctacagcctgaagatactt
Actb	NM_007393.5	For: ctctagacttcgagcaggagatgg<br>Rev: atgccacaggattccatacccaaga
Adgre1	NM_010130.5	For: ggaggacttctccaagcctatt<br>Rev: aggcctctcagacttctgctt
Arg1	NM_007482.3	For: ggaaagccaatgaagagctg<br>Rev: ctggttgtcaggggagtgtt
Cd163	NM_001170395.1	For: gtgctggatctcctggttgt<br>Rev: tcattcatgctccagccgtt
Cd68	NM_001291058.1	For: aggaccgcttatagcccaag<br>Rev: atttccgtgactggtggtg
Fpn1	NM_009096.3	For: acccatccccatagtctctgt<br>Rev: accgtcaaatcaaaggacca
Fth1	NM_010239.2	For: tggagttgtatgcctcctacg<br>Rev: tggagaaagtatttggcaaagtt
Ftl1	NM_010240.2	For: agcgtctcctcgagtttcagaa<br>Rev: gggttttaccccattcatcttg
Gpx4	NM_008162.4	For: gcaaccagtttgggaggcaggag<br>Rev: cctccatgggaccatagcgcttc
Hamp1	NM_032541.2	For: ctgagcagcaccacctatctc<br>Rev: tggctctaggctatgttttgc
Hmox1	NM_010442.2	For: gtgatggagcgtccacagc<br>Rev: ttggtggcctccttcaagg
Il1r1	NM_001123382.2	For: attgttgaacatcgccactg<br>Rev: aaatgagccccagtagcactt
Ireb2	NM_022655.3	For: tgaagaaacggacctgctct<br>Rev: gctcacatccaaccacctct
Mcpt2	NM_008571.2	For: ttcaccactaagaacggttcg<br>Rev: ctccaaggatgacactgatttca
Mcpt6	NM_010781.3	For: tgctgtgtgctgcaaatacc<br>Rev: cccttcactttgcagacca
Mrc1	NM_008625.2	For: tggaggctgattacgagcag<br>Rev: atgccagggtcacctttcag
Nos2	NM_010927.4	For: ctttgccacggacgagac<br>Rev: tcattgtactctgagggctgac
Slc11a2	NM_001146161.1	For: ggctcctgggatatggagtc<br>Rev: tctgtgctcttagaataggattcg
Tf	NM_133977.2	For: cgcagtcctcttgagaaagc<br>Rev: agcctgggcacagttgac

Suppl. Table 2: List of Antibodies used in Western Blot Analysis

Name	Catalog No.	Dilution	Clonality/Host	Company
**Primary Antibodies**				
Anti- Glutathione Peroxidase 4	ab125066	1:1,000	mono, rabbit	Abcam, Cambridge, UK
CD68 (KP1)	sc-20060	1:1,000	mono, mouse	Santa Cruz Biotechnology, Dallas, TX, USA
Ferritin heavy chain (B-12)	sc-376594	1:500	mono, mouse	Santa Cruz Biotechnology
Ferritin light chain (D-1)	sc-390558	1:500	mono, mouse	Santa Cruz Biotechnology
GAPDH	sc-32233	1:1,000	mono, mouse	Santa Cruz Biotechnology
Heme Oxygenase 1 (F-4)	sc-390991	1:1,000	mono, mouse	Santa Cruz Biotechnology
Transferrin	17435-1-AP	1:1,000	poly, rabbit	ProteinTech® GmbH, Planegg-Martinsried, Germany
β-actin	A5441	1:10,000	mono, mouse	Sigma-Aldrich, Taufkirchen, Germany
**Secondary Antibodies**				
goat anti-rabbit IgG (H+L), HRP	31460	1:5,000	poly, goat	Thermo Fisher Scientific, Waltham, MA, USA
goat anti-mouse IgG (H+L), HRP	31430	1:5,000	poly, goat	Thermo Fisher Scientific

Suppl. Table 3: Concentrations of Elements in the Ovaries measured by LA-ICP-MS

	^{23}Na (µg/g)		^{24}Mg (µg/g)		^{31}P (µg/g)	
	**Mean**	**SD**	**Mean**	**SD**	**Mean**	**SD**
**WT**	3838	1213	387.2	149	8167.2	2766
Esr1$^{-/-}$	6822	1589	415.8	119	11640.4	2277
Statistics	*p*=0.0103*		*p*=0.7457		*p*=0.0620	
	^{34}S (µg/g)		^{39}K (µg/g)		^{44}Ca (µg/g)	
	**Mean**	**SD**	**Mean**	**SD**	**Mean**	**SD**
**WT**	879.8	226	2976.0	742	28.4	15
Esr1$^{-/-}$	1050.4	233	4133.2	1405	38.06	12
Statistics	*p*=0.2742		*p*=0.1420		*p*=0.2852	
	^{52}Cr (µg/g)		^{55}Mn (µg/g)		^{56}Fe (µg/g)	
	**Mean**	**SD**	**Mean**	**SD**	**Mean**	**SD**
**WT**	0.3	0.01	0.4	0.2	148	63
Esr1$^{-/-}$	0.2692	0.02	0.3276	0.0	595	189
Statistics	*p*=0.0166*		*p*=0.3714		*p*=0.001**	
	^{63}Cu (µg/g)		^{64}Zn (µg/g)			
	**Mean**	**SD**	**Mean**	**SD**		
**WT**	1.4	1	29.9	9		
Esr1$^{-/-}$	1.841	0	27.92	4		
Statistics	*p*=0.2148		*p*=0.6814			
