# Supplementary material for: Ovaries of estrogen receptor 1-deficient mice show iron overload and signs of aging
Source: Front Endocrinol (Lausanne). 2024 Feb 23;15:1325386. doi: 10.3389/fendo.2024.1325386 (PMC10920212; doi:10.3389/fendo.2024.1325386)
Supplement: Supplementary file 2 [file Presentation_1.pptx]

## Slide 1
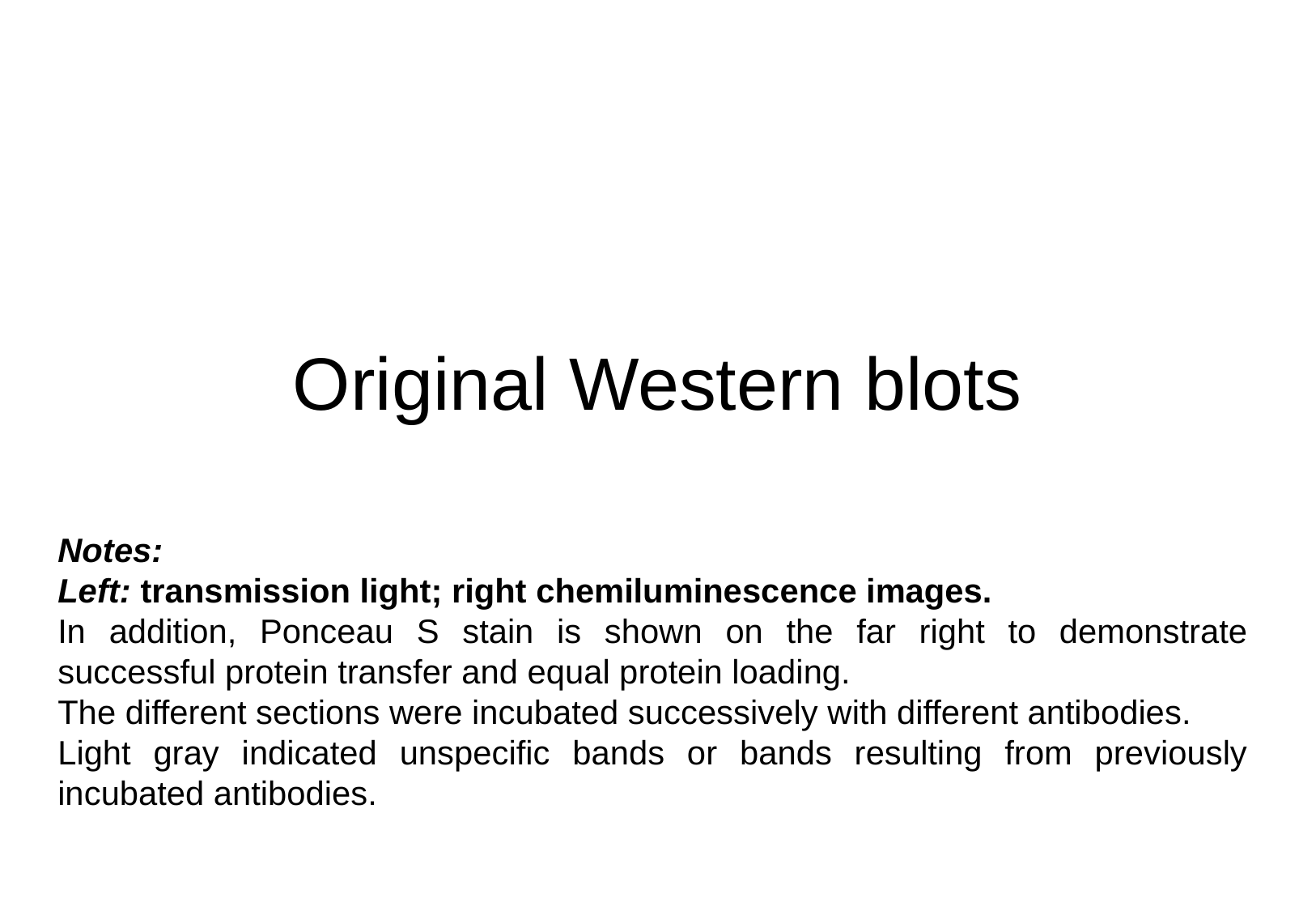

# Original Western blots
Notes:
Left: transmission light; right chemiluminescence images.
In addition, Ponceau S stain is shown on the far right to demonstrate successful protein transfer and equal protein loading.
The different sections were incubated successively with different antibodies.
Light gray indicated unspecific bands or bands resulting from previously incubated antibodies.

## Slide 2
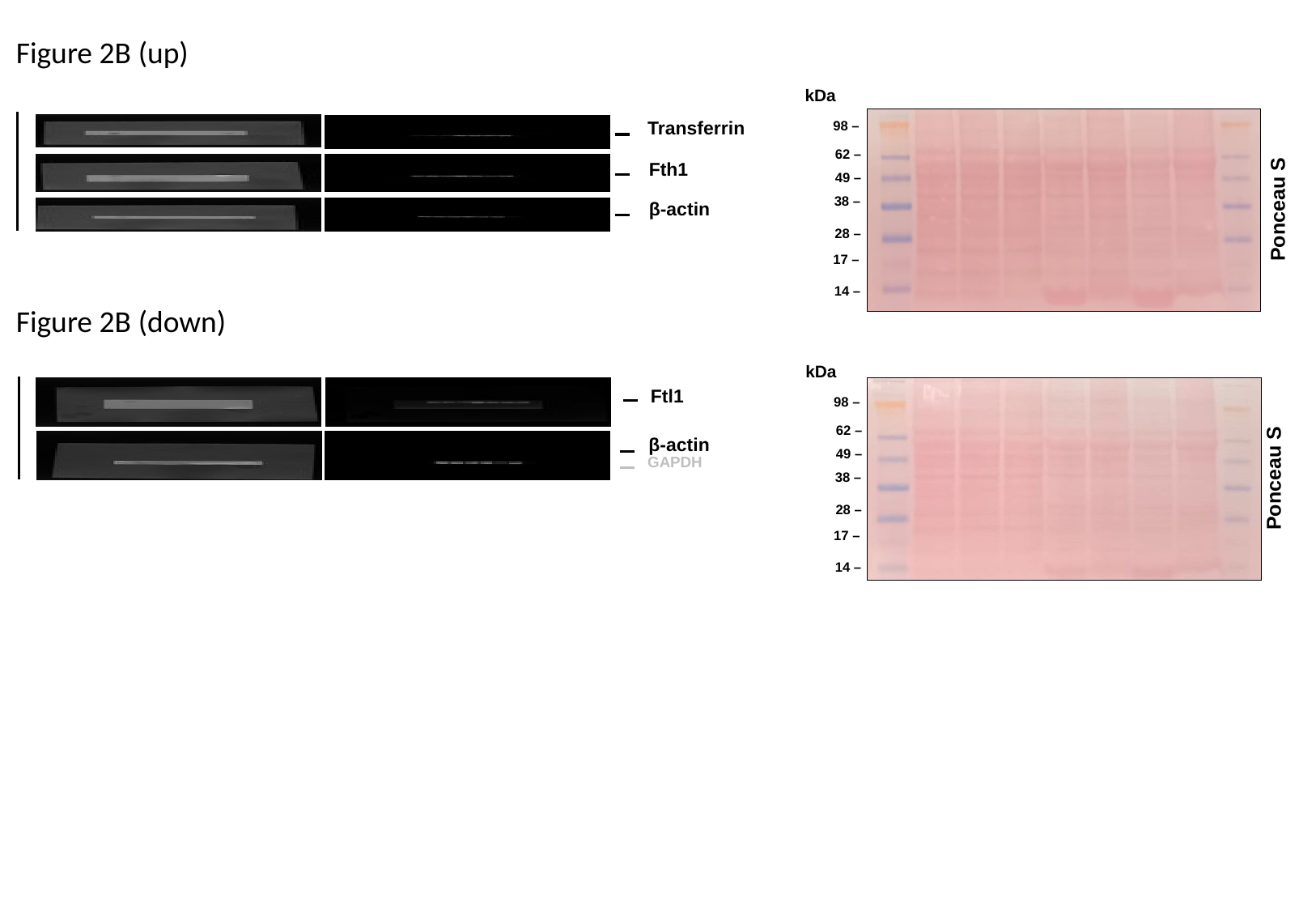

Figure 2B (up)
Transferrin
kDa
Fth1
Ponceau S
98 –
62 –
49 –
β-actin
38 –
28 –
17 –
14 –
Figure 2B (down)
Ftl1
kDa
Ponceau S
98 –
β-actin
62 –
GAPDH
49 –
38 –
28 –
17 –
14 –

## Slide 3
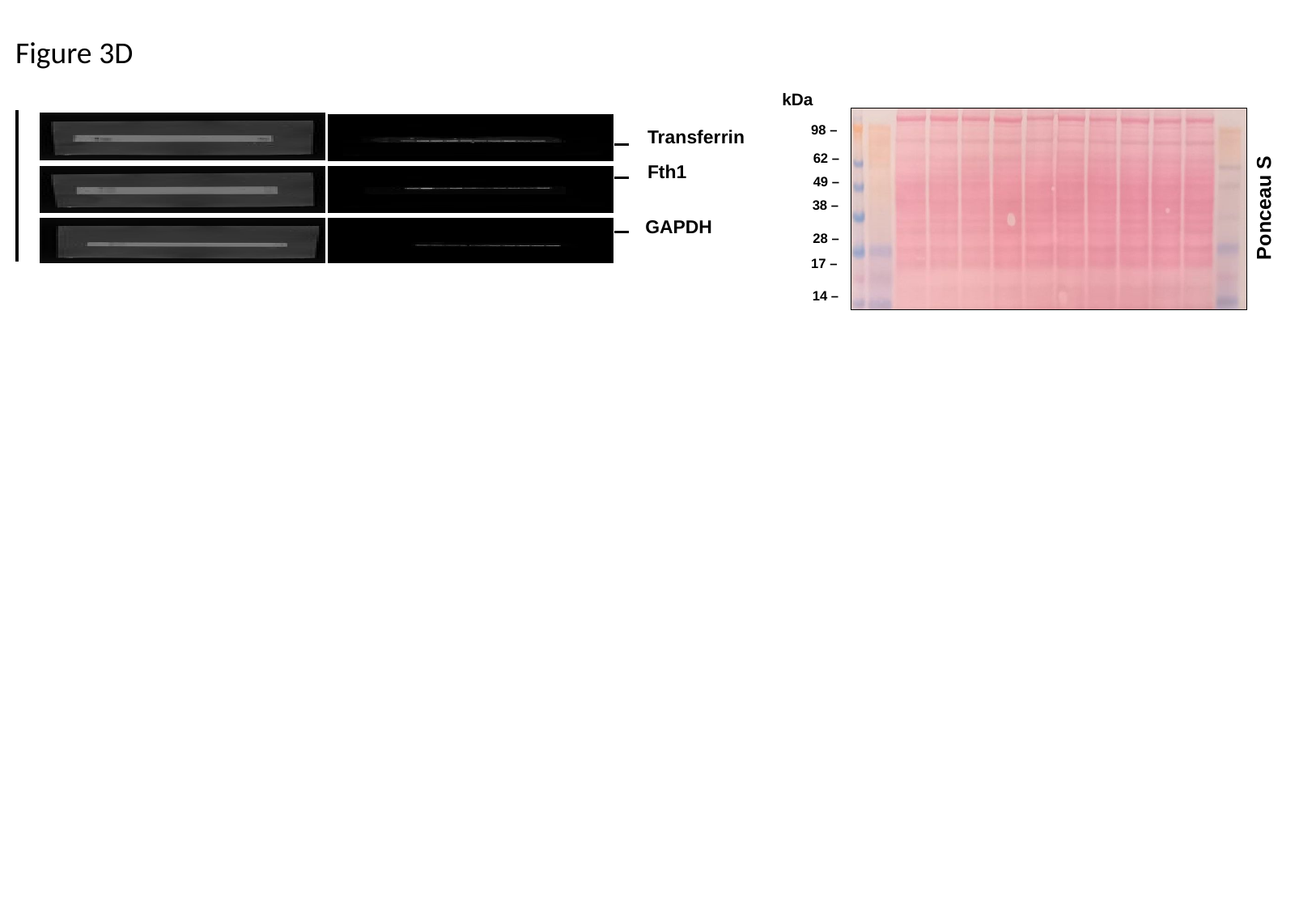

Figure 3D
Transferrin
kDa
Ponceau S
Fth1
98 –
62 –
49 –
GAPDH
38 –
28 –
17 –
14 –

## Slide 4
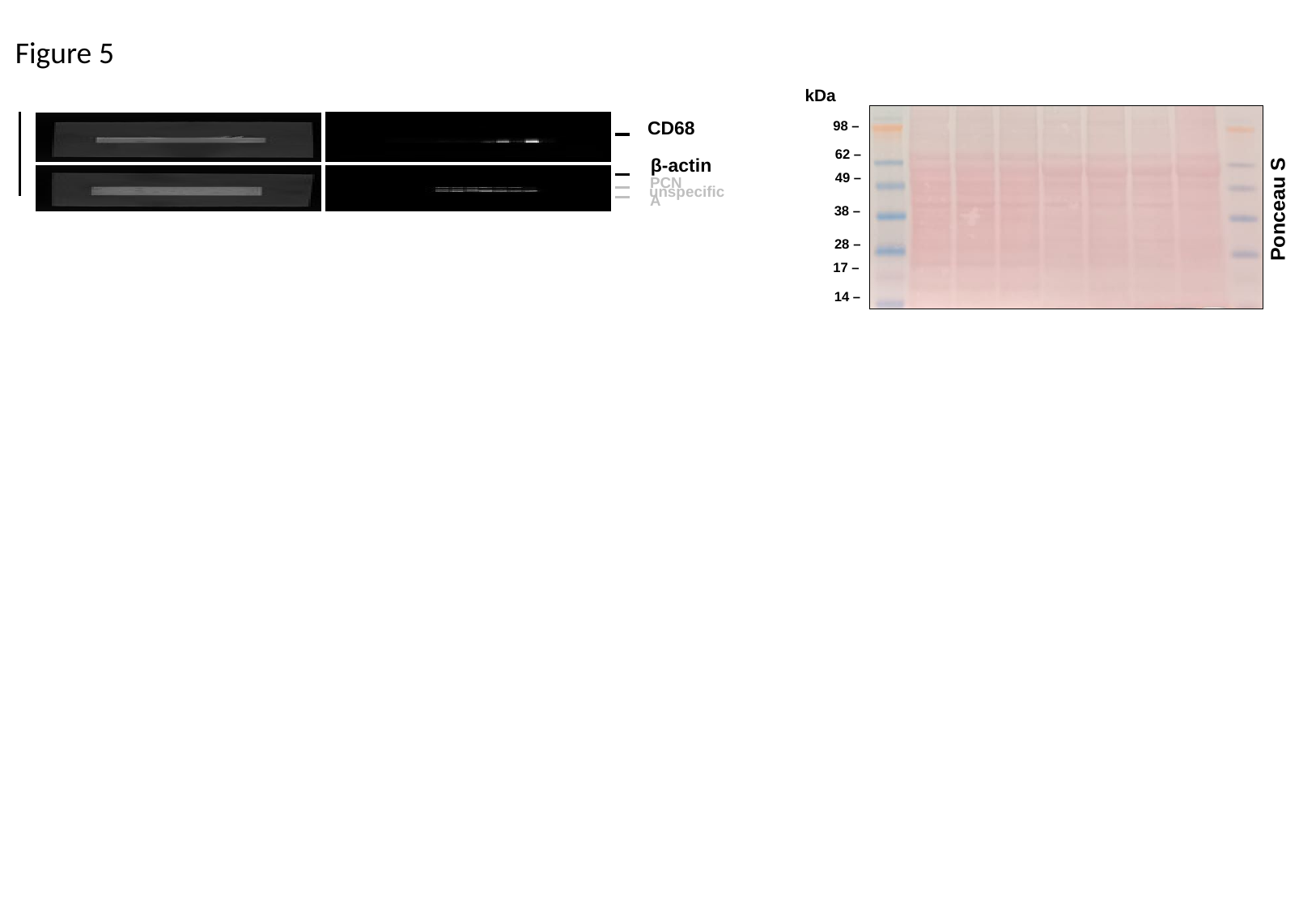

Figure 5
CD68
kDa
Ponceau S
98 –
β-actin
unspecific
62 –
PCNA
49 –
38 –
28 –
17 –
14 –

## Slide 5
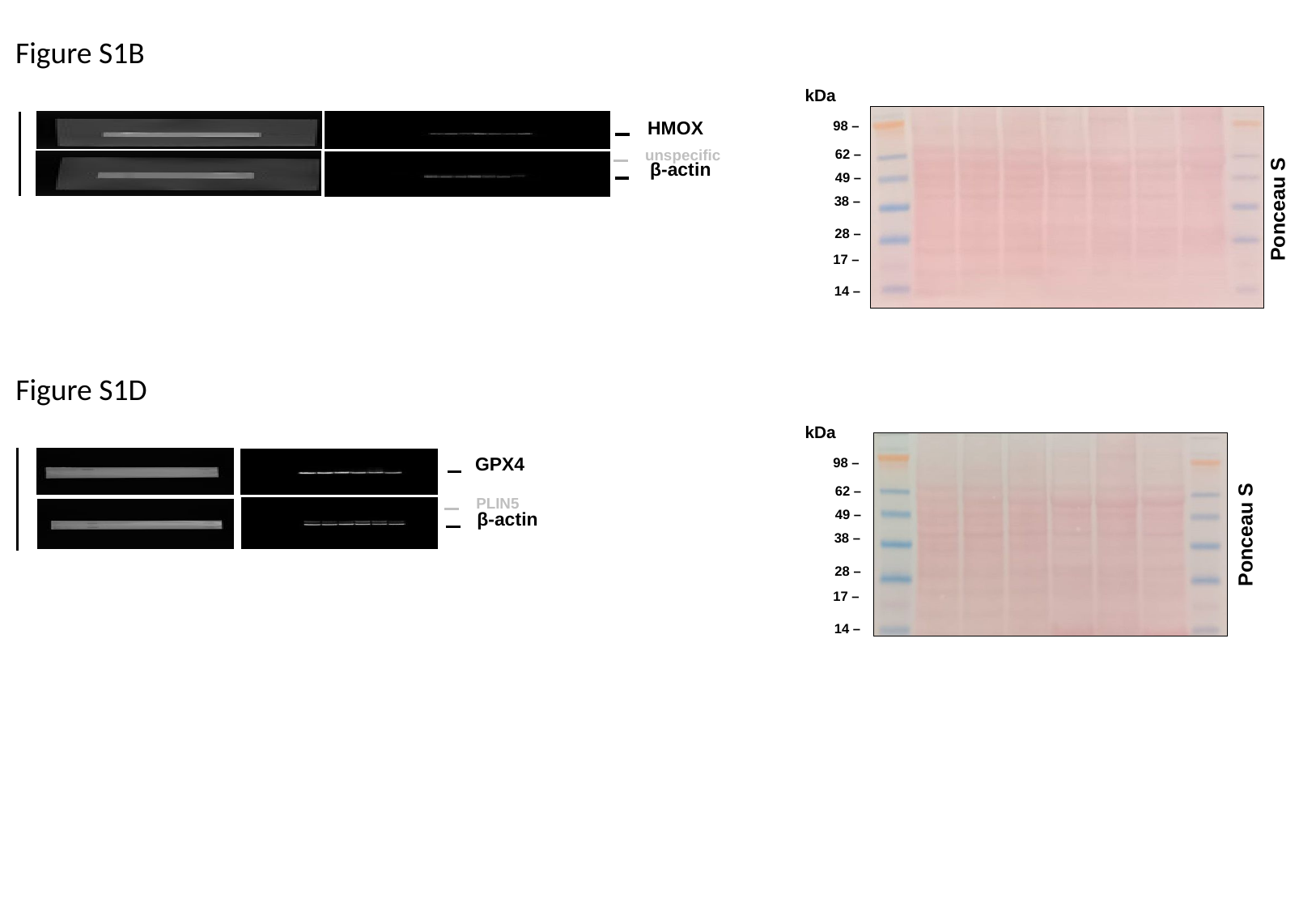

Figure S1B
HMOX
kDa
Ponceau S
unspecific
98 –
β-actin
62 –
49 –
38 –
28 –
17 –
14 –
Figure S1D
GPX4
kDa
Ponceau S
98 –
PLIN5
β-actin
62 –
49 –
38 –
28 –
17 –
14 –

## Slide 6
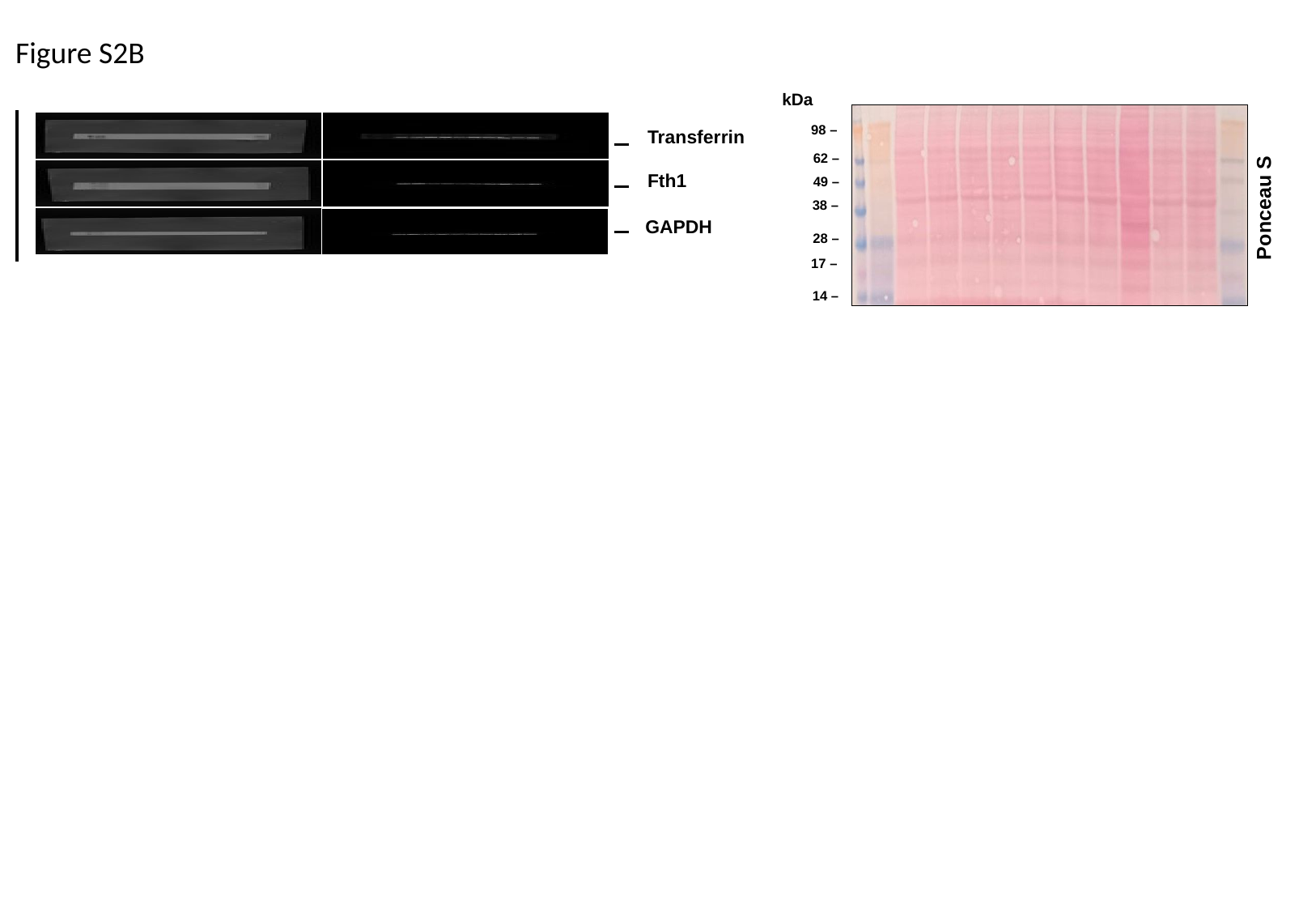

Figure S2B
Transferrin
kDa
Ponceau S
98 –
Fth1
62 –
49 –
GAPDH
38 –
28 –
17 –
14 –
